# A neuro-inspired computational model of life-long learning and catastrophic interference, mimicking hippocampus novelty-based dopamine modulation and lateral inhibitory plasticity

**DOI:** 10.3389/fncom.2022.954847

**Published:** 2022-09-09

**Authors:** Pierangelo Afferni, Federico Cascino-Milani, Andrea Mattera, Gianluca Baldassarre

**Affiliations:** ^1^Department of Engineering, Campus Bio-Medico University, Rome, Italy; ^2^Department of Genetics and Neurobiology, Julius-Maximilians Universität Würzburg, Würzburg, Germany; ^3^Laboratory of Embodied Natural and Artificial Intelligence, Institute of Cognitive Sciences and Technologies, National Research Council, Rome, Italy

**Keywords:** life-long learning, catastrophic forgetting, novelty detection, dopamine, inhibitory synaptic plasticity, hippocampus

## Abstract

The human brain has a remarkable lifelong learning capability to acquire new experiences while retaining previously acquired information. Several hypotheses have been proposed to explain this capability, but the underlying mechanisms are still unclear. Here, we propose a neuro-inspired firing-rate computational model involving the hippocampus and surrounding areas, that encompasses two key mechanisms possibly underlying this capability. The first is based on signals encoded by the neuromodulator dopamine, which is released by novel stimuli and enhances plasticity only when needed. The second is based on a homeostatic plasticity mechanism that involves the lateral inhibitory connections of the pyramidal neurons of the hippocampus. These mechanisms tend to protect neurons that have already been heavily employed in encoding previous experiences. The model was tested with images from the MNIST machine learning dataset, and with more naturalistic images, for its ability to mitigate catastrophic interference in lifelong learning. The results show that the proposed biologically grounded mechanisms can effectively enhance the learning of new stimuli while protecting previously acquired knowledge. The proposed mechanisms could be investigated in future empirical animal experiments and inspire machine learning models.

## 1. Introduction

The human brain has an impressive life-long learning capability that allows the acquisition of new experiences while retaining previously stored information (Parisi et al., 2019). Even though the new information may retroactively interfere with that already acquired (Barnes and Underwood, 1959), the human brain only rarely undergoes a severe forgetting of old information (Pallier et al., 2003; Parisi et al., 2019). On the other hand, artificial neural networks tend to forget previously acquired information when learning new patterns (McCloskey and Cohen, 1989; French, 1999). This phenomenon has a typical dynamic that differs from human retroactive interference and is more similar to retrograde amnesia (McCloskey and Cohen, 1989; Parisi et al., 2019). Several hypotheses have been proposed on how the brain avoids catastrophic forgetting.

A proposal to protect old information in artificial neural networks is the Elastic Weight Consolidation mechanism (Kirkpatrick et al., 2017). This hypothesis is inspired by some notable properties of the brain. First, it has been shown that two different motor tasks produce calcium transients and spine enlargement in dendritic spines located on separate dendritic branches of the motor cortex layer-5 neurons (Cichon and Gan, 2015). This localized activation and potentiation of dendritic spines can be abolished through the inhibition of somatostatin-expressing interneurons. When this happens, the two tasks activate dendritic spines on the same branches and the learning of the second task interferes with the retention of the first one. This suggests that interneurons are responsible for spatial segregation of task-related information on different synapses, possibly to avoid catastrophic forgetting. Moreover, task-specific potentiated dendritic spines represent the physical trace of the acquired skill (Yang et al., 2009) and a large portion of them (~70%) are stable for months after training (Hayashi-Takagi et al., 2015). On the basis of this, the Elastic Weight Consolidation algorithm constraints the variability of the most important parameters for the performance of a certain task, while learning other tasks. By employing this biologically grounded strategy, the algorithm achieves a localistic connection stability in deep neural networks that ensures protection of the old acquired tasks (Kirkpatrick et al., 2017). The work on Elastic Weight Consolidation demonstrates that in a deep neural network the information must be stored in localized connections in order to be protected. We considered this aspect in our work. However, this model has been implemented only for supervised learning and reinforcement learning, but not for unsupervised learning. Moreover, the deep neural network employed by Kirkpatrick et al. (2017) is not biologically plausible.

A recent proposal to ameliorate catastrophic interference is that a novelty signal could be generated and used to protect previous memories. Both the hippocampus and different areas of the cortex, for example, the perirhinal and prefrontal cortices and the ventral visual stream regions, are highly responsive to novelty (Parker et al., 1998; Kishiyama et al., 2009; Barto et al., 2013; Kafkas and Montaldi, 2014, 2018). Concerning the hippocampus, a theory has been proposed on how such novelty is detected and used by means of the neuromodulator dopamine to control long-term potentiation (LTP; Lisman and Grace, 2005). Experimental results demonstrate that novel, but not familiar stimuli, induce a burst of activation in dopamine-releasing neurons in the ventral tegmental area (VTA; Steinfels et al., 1983; Ljungberg et al., 1992). The pathway leading to the novelty-induced VTA activation passes throught the subiculum, the nucleus accumbens, and the ventral pallidum (Legault et al., 2000; Floresco et al., 2001, 2003; Legault and Wise, 2001). The dopamine released by the VTA is sent back to the hippocampus, thus instructing it to store the new information through the consolidation of the late phase of LTP (Lisman and Grace, 2005). We have implemented in our model an original mechanism to detect novelty and to automatically modulate dopamine release in the hippocampus, inspired by the work of Lisman and Grace (2005). Another recent work attempts to model this mechanism by introducing a dopaminergic contribution, which increases or decreases inversely to the level of neuronal activation, to prevent a neuron that has already learned from becoming dominant over other neurons when the network is exposed to new stimuli (Allred and Roy, 2020). This model represents a seminal work that, following the principle of localized learning, exploits novelty to face catastrophic interference in an unsupervised learning context. However, the model does not have a biological correspondence for various aspects it incorporates, such as the use of a variable dopaminergic weight for each neuron, the reset of the network before learning a new image, and the change of the learning rate of the first neuron that fires after image perception to protect it from further learning.

A mechanism also considered here is based on the modulation of the excitation/inhibition balance *via* interneurons. It has been observed that high-frequency stimulation of the CA3 to CA1 Schaffer pathway induces LTP in excitatory neurons, and in parallel increases the intrinsic excitability of interneurons to adjust the feedforward inhibition (Campanac et al., 2013). Besides excitability, several different mechanisms of plasticity have been identified in interneurons that potentially affect the excitation/inhibition balance in the network (Hartmann et al., 2008; Vogels et al., 2013; Chevaleyre and Piskorowski, 2014). Computational modeling suggests that inhibitory plasticity is a homeostatic mechanism, allowing new information to be acquired through Hebbian learning and retrieved at a later time while maintaining network stability (Vogels et al., 2011). Furthermore, it has been shown that inhibitory plasticity can provide a mechanism for the preservation of place fields when returning to a first evironment after exposure to a second environment, thus protecting hippocampal place cells from interference (Udakis et al., 2020). The model of Udakis et al. (2020) is the first to propose that inhibitory plasticity can protect the acquired knowledge from succeeding experiences. However, while relying on biologically grounded learning rules for inhibitory plasticity of parvalbumin and somatostatin interneurons, it models the hippocampus place cells with a very simplified architecture and input patterns.

Here, we present a new computational model, encompassing various architectural and functional elements of the brain's hippocampus-dopamine system, that proposes and studies an original combination of the effects of dopaminergic signaling (Lisman and Grace, 2005) and pyramidal neuron inhibitory synapses plasticity (Udakis et al., 2020) in order to minimize the phenomenon of interference during continuous learning of different types of images. Specifically, the model is able to enhance learning when dopamine release is triggered by novelty signals generated intrinsically by the network itself, and to stop dopamine release, and thus learning, in the case of familiar stimuli. Furthermore, inhibitory synaptic plasticity produces a homeostatic effect on the activity of neurons that are learning a given piece of information. This prevents them from acquiring dominant excitability and subsequently coding a multiplicity of different patterns. In this way, the model achieves the separation of population codes representing multiple memory traces, which is necessary to avoid catastrophic interference (Kirkpatrick et al., 2017).

The two mechanisms cooperate in different ways to avoid catastrophic forgetting. We highlighted this by testing the model with different types of input images. In particular, we used two image datasets having different characteristics: (a) the well-known MNIST dataset containing images representing handwritten numerical characters; (b) a dataset of images representing naturalistic landscapes taken from the scenery of Lake Como. These datasets allowed us to test both the normal functioning model and the model missing either one of the two mechanisms or both. In experiments, we used a common procedure to measure catastrophic interference, specifically the rate of recognition after learning a variable number of new tasks (French, 1999; Allred and Roy, 2020). Moreover, we employed the MNIST dataset to measure the generalization ability of the system using test images different from training images.

The model was also tested with a second type of experiment involving a more realistic continuous learning challenge (lifelong learning test) that mimics the tests proposed by Udakis et al. (2020). These experiments simulated an agent that *fully learns* two different environments (datasets) in succession and then returns to the first environment. These experiments probed the stability of the representations generated by the model based on both mechanisms: novelty-activated dopaminergic learning and homeostatic plasticity of the lateral inhibitory connection.

The tests show the effectiveness of the two biologically inspired mechanisms to face catastrophic interference in different conditions (two types of tests) and with different datasets (MNIST images organized by category, and Lake Como naturalistic images). In the catastrophic interference test, lateral inhibitory plasticity seems to play a more important role in preventing catastrophic forgetting with the MINST dataset; in contrast, dopamine modulation following the novelty signal seems more important with the Lake Como images. In the lifelong learning experiment, both mechanisms are necessary to avoid instability of previously acquired experiences with MNIST images, whereas dopamine is sufficient to ensure stability with Lake Como images. The results also show how the two mechanisms can avoid catastrophic forgetting by keeping the neuronal populations that encode different tasks separated, similar to what is otherwise achieved with machine learning methods (Kirkpatrick et al., 2017).

## 2. Materials and methods

### 2.1. The neural network model

**Figure 1:**
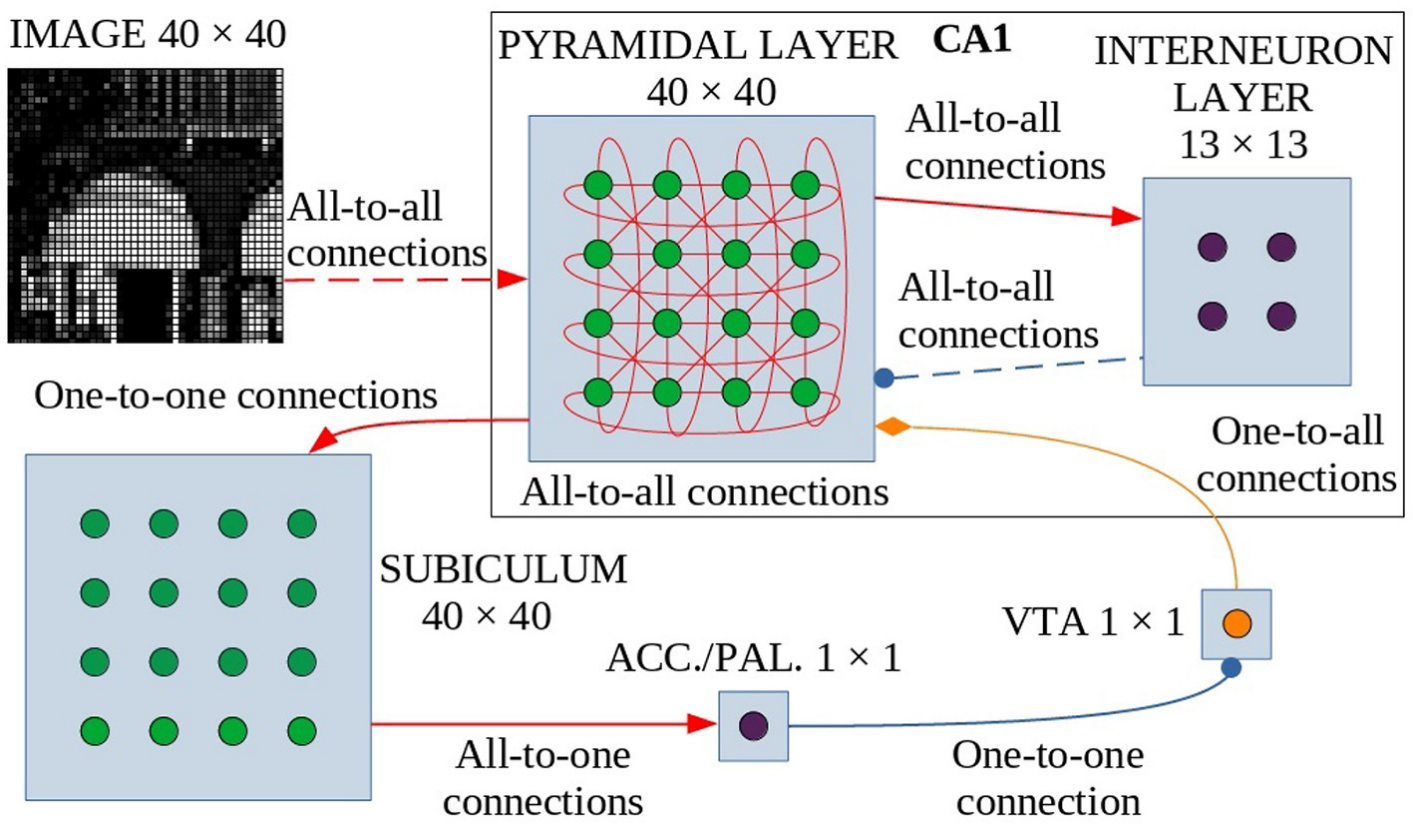
Model architecture. Green circles are excitatory neurons, purple circles are inhibitory neurons and the orange circle indicates dopaminergic neurons. Red arrows are the excitatory connections, the blue connectors with a circle indicate the inhibitory connections, and the orange one with the diamond represents the dopaminergic connection. The dashed lines indicate synapses that change their weight during learning. These synapses undergo dopamine-based learning. Input is connected all-to-all with the pyramidal layer of CA1, a population of interconnected excitatory neurons (without self-connections). Within CA1, pyramidal neurons excite inhibitory interneurons which in turn inhibit pyramidal neurons. The strength of all other connections in CA1 depends on a distance-based function (see text for details). The CA1 pyramidal layer is also connected with the Subiculum through one-to-one connections: when one or more neurons of the pyramidal layer reach a sufficient activation level, they activate the corresponding neurons in the Subiculum. The activation of at least one neuron is necessary and sufficient to trigger the inhibitory neuron of Accumbens/V.Pallidum to turn off the dopaminergic neuron in VTA, thus stopping learning. ACC./PAL. indicates the Accumbens/V. Pallidum.

The model is formed by six neuronal layers that perform specific functions (Figure 1), where the core layer is represented by the pyramidal layer of the CA1 region of the hippocampus. These neuronal layers are an abstraction of four regions of the hippocampus-midbrain system (Lisman and Grace, 2005).

The model receives an external visual signal represented by an image composed of gray-tone pixels. The neurons of the first layer of the model (input layer, *N* = 1,600 neurons, Supplementary Table 2) encode the pixels of the observed image, each activating in proportion to the gray level of the image pixels (see Supplementary Methods: Input pre-processing).

The CA1 region is formed by a layer of excitatory neurons (*N* = 1,600, Supplementary Table 2), representing pyramidal cells, and a layer of inhibitory neurons (*N* = 169, Supplementary Table 2), representing interneurons. The network is designed to function as a SOM (Self-Organizing Map): it selects a few neurons that respond specifically to an input, according to the “winner-takes-all” paradigm, due to connectivity between layers (which produces a neuronal field resembling a Mexican hat, as described in Section 2.2 and Supplementary Figure 1) and a specific learning rule that can take into account the dynamic properties of the network. When an applied external input is received, after an initial transient phase, the network self-organizes through plastic connections between the input layer and the CA1. This process brings activation to a steady state in which a small group of locally connected pyramidal neurons are more active than the others. This “winning population” of neurons encodes the input pattern. However, the architecture of our model is biologically more plausible than a standard SOM, as it is composed of recurrent connections involving excitatory and inhibitory neurons, which generate a dynamic neural competition. Below, and in particular in Section 2.2, we describe the characteristics of the connection scheme adopted in the model and how it was modeled in analogy to biological data (Bezaire et al., 2016). In addition, it is important to note that our model, in order to have a more natural behavior and unlike others (Allred and Roy, 2016), does not reset the state of the CA1 network to the initial condition before each presentation of a new image.

The output of CA1 is routed in a multisynaptic pathway through three different areas, Subiculum—Accumbens/V. Pallidum—VTA, which implements dopamine modulation based on novelty detection (Lisman and Grace, 2005). Subiculum neurons (*N* = 1,600, Supplementary Table 2) have a high activation threshold, so a neuron within this area fires to the Accumbens/V. Pallidum only when the corresponding CA1 pyramidal neuron is sufficiently active. To simplify, we have represented the Accumbens/V. Pallidum with a single neuron and also the VTA with a single dopaminergic neuron, assuming for the purpose of this study that all neurons in those areas behave homogeneously.

When VTA fires, dopamine is released on CA1 pyramidal neurons, allowing them to learn. Conversely, when VTA receives inhibitory input it stops releasing dopamine. When a novel image is presented to the network there is a low correlation between the input pattern and each of the connection weights groups targeting distinct CA1 neurons, and as a consequence, the CA1 neurons have a low activation. This condition can be interpreted as the fact that the system is classifying the stimulus as “novel”. When the input-to-pyramidal connection weights progressively change by learning, the activation of the winning population of CA1 neurons grows. This can be interpreted as the fact that the system is classifying the stimulus as “familiar”. When the activation of the peak neuron within the winning CA1 population activates the corresponding neuron of the Subiculum above its threshold, this activates the neuron in Accumbens/V. Pallidum, which, in turn, inhibits the dopaminergic neuron in VTA. Familiar input patterns thus stop learning. After learning, the network is able to discriminate between different images because the information from each image is stored locally in the synaptic weights of separate or possibly slightly overlapping populations of winning neurons.

#### 2.1.1. Implementation details of the model

The model was implemented in Python 3.5.10, using NumPy libraries for scientific computing, and PyQt5 for the graphical interface. The followings software packages were used to set the python environment required to execute the model: matplotlib 3.0.3, numpy 1.16.3, numpydoc 0.8.0, Pillow 6.1.0, PyQt5 5.12, PyQt5-sip 4.19.17, paragraph 0.11.0, QtAwesome 0.5.6, qt-console 4.4.3, QtPy 1.6.0, Jason 3.2.0, mnist 0.2.2, python-mnist 0.7.

The code and the input datasets of MNIST and Lake Como images can be found at: https://github.com/pafferni/A-neuro-inspired-computational-model-of-life-long-learning-and-catastrophic-interference.

### 2.2. CA1: Micro-architecture and biological basis

We now explain in more detail the specific connectivity of CA1 in our model, the core part of the model, and show how it reflects some key qualitative features of the CA1 connectivity observed in the biological brain.

Considering that the model represents CA1, we used a ratio of 9 to 1 between the number of pyramidal neurons and the number of generic interneurons (Bezaire and Soltesz, 2013). The model is parametric and could be easily adapted to be used in future work to simulate neural networks of different sizes and ratios between pyramidal neurons and interneurons and different connectomics. For example, the model could be applied to study the CA3 with a ratio of 4:1, although the parameters and connectomics of the model should be adapted to reflect the different structures of this area (Guzman et al., 2016). The number of neurons forming each neuronal layer of the current model are reported in Supplementary Table 2.

Each input neuron is then connected to all pyramidal neurons of the CA1 region through all-to-all connections. The input-to-pyramidal connection weights $w_{post,pre}^{INP}$ (with *pre* and *post* representing respectively the pre-synaptic and post-synaptic neurons) are initially set with the equation $w_{post,pre}^{INP}=Normalize\left[random\right]$, where *random* is a random value uniformly drawn in the interval [0,1) and *Normalize*[] computes the *L*_2_ norm. Subsequently, these weights can be changed as a result of learning according to the learning rule described in the Section 2.4 by Equation (9).

The CA1 neurons in the model lie on a 2D square map to capture as a first approximation some relevant aspects of the spatial organization of neurons in the CA1 hippocampus area. The neurons that are on the four sides of the map edge have connections with those on the opposite side and are considered close in space as if the two opposite sides were adjacent to avoid the edge effect.

To simulate the natural probability of connection within the CA1 area, we assumed that the lateral connectivity of the excitatory pyramidal neurons in CA1 and inhibitory neurons are generated by a random process in which the weight has a value calculated as the product of: (a) a factor dependent on the distance, within the neural 2D layer, between the two connected units; (b) a random number generated with a uniform distribution in the interval [0, 1). Supplementary Figure 1 indicates the values of the distance-dependent factor used to calculate the connection weights, for the different types of connections. The values of the pyramidal-to-pyramidal connections and of the pyramidal-to-interneuron connections (not shown in the figure) follow a similar function of distance represented by a “narrow” Gaussian curve, obtained respectively from Equation (1) with different values of the parameter *s*_1_. The values of the interneuron-to-pyramidal (inhibitory) connections follow a function of distance represented by a large Mexican-hat curve, obtained from Equation (2). Supplementary Figure 2 shows, for the three types of lateral connections, examples of the connections referring to one CA1 neuron, having a connection weight above a threshold of 0.04. Supplementary Figure 3 shows the above-threshold connection frequencies as a function of the distance, measured after the network has been generated. For comparison, Supplementary Figure 4 shows the connection frequencies extrapolated from the experimental data for the CA1 of the rat, as reported in Bezaire et al. (2016).

We used the following equation to generate the weight $w_{post,pre}^{PP}$ of the lateral pyramidal-to-pyramidal connection, converging to one CA1 pyramidal neuron (parameter *s*_1_ is in Supplementary Table 1, Layer: exc-exc):


(1) $$\begin{array}{r} w_{post,pre}^{PP}=Normalize\left[e^{-d_{post,pre}^{2}\cdot s_{1}}\cdot random\right] \end{array}$$

where *d*_*post, pre*_ is the distance between the pre-synaptic neuron and post-synaptic neuron, the factor *random* represents a random value drawn from a uniform distribution over the interval [0, 1), and the function *Normalize*[] performs the *L*_2_ norm of the vector of weights *w*.

The same equation, but with a different value for parameter *s*_1_, was used to generate the weight $w_{post,pre}^{PI}$ of each pyramidal-to-interneuron connection (see the parameter *s*_1_ value in Supplementary Table 1, Layer: exc-inh).

The following equation was instead used to generate the weight $w_{post,pre}^{IP}$ of the interneuron-to-pyramidal connection converging to a CA1 pyramidal neuron (parameters *s*_1_, *s*_2_ are in Supplementary Table 1, Layer: inh-exc):


(2) $$\begin{array}{r} w_{post,pre}^{IP}=Normalize\left[0.5\cdot \left(e^{-d_{post,pre}^{2}\cdot s_{1}}-e^{-d_{post,pre}^{2}\cdot s_{2}}\right)\cdot random\right] \end{array}$$

The formulas using the distance between neurons employed as unitary measure the minimum distance between two neighboring neurons.

The Subiculum is formed by a layer of excitatory neurons receiving one-to-one afferent connections (weights = 1) from the CA1 excitatory neurons. The Subiculum neurons have all-to-one excitatory efferent connections (weights = 1) to the Accumbens/V. Pallidum single neuron which in turn has an inhibitory connection (weight = −1) to the VTA single neuron, having an on/off type activation.

### 2.3. Implementation of the neurons in the model

Each pyramidal neuron in the model is implemented as a leaky integrate-and-fire neuron. The variation of the pyramidal neuron inner potential $u_{post}^{P}\left(t\right)$ at time *t*, depending on the time constant parameter τ (Supplementary Table 3), is obtained as follows:


(3) $$\begin{array}{r} \tau \Delta u_{post}^{P}=-u_{post}^{P}+SI+LE+LI+Noise \end{array}$$

The components of the Equation (3) are computed as follows:

$SI=\underset{pre}{\sum }\left(w_{post,pre}^{INP}\cdot a_{pre}^{INP}\left(t\right)\right)$, represents the sensory input, where $a_{pre}^{INP}\left(t\right)$ is the activation of a pre-synaptic input neuron at time *t*, and $w_{post,pre}^{INP}$ is the connection weight between the input pre-synaptic neuron and the post-synaptic pyramidal neuron;

$LE=\underset{pre}{\sum }\left(w_{post,pre}^{PP}\cdot a_{pre}^{P}\left(t\right)\right)$, represents the lateral excitation, where $a_{pre}^{P}\left(t\right)$ is the activation of another pre-synaptic CA1 pyramidal neuron, and $w_{post,pre}^{PP}$ is the connection weight between the pre- and post-synaptic pyramidal neurons;

$LI=\underset{pre}{\sum }\left(w_{post,pre}^{IP}\cdot a_{pre}^{I}\left(t\right)\right)$, represents lateral inhibition, where $a_{pre}^{I}\left(t\right)$ is the activation of a pre-synaptic interneuron, and $w_{post,pre}^{IP}$ is the connection weight between the pre-synaptic interneuron and the post-synaptic pyramidal neuron;

*Noise* = λ·*rand*·İ_*post*_ where *rand* is a random value uniformly drawn from interval [0, 1), λ is the noise level (Supplementary Table 4), and İ_*post*_ is the sum of all absolute values of the time derivatives of the excitatory synaptic potentials (each synaptic potential being $w_{post,pre}^{INP}\cdot a_{pre}^{INP}$) and is used to lower the noise when the neuron input signals stabilize.

Here, all terms of the type *a*(*t*) denote activation of neurons and for both pyramidal neurons ($a_{pre}^{P}\left(t\right)$) and interneurons ($a_{pre}^{I}\left(t\right)$) they are obtained by applying the neuronal gain function described in Equation (4) to the activation inner potential *u*(*t*) of the neuron: *a*(*t*) = *fu*(*t*). The initial values, at time zero (start of model execution), of the potential *u*(*t*) of all neurons are set to zero.

The neuron gain function is as follows:


(4) $$f(x)=\left\{ \begin{array}{l} 0\text{ for }x=<0\\ min\left[1,\;sinh(x)\cdot h+0.008\right]\text{ for }x>0 \end{array}\right.$$

where the function *min* ensures that f(x) is maximum 1. Each CA1 interneuron in the model is also implemented as a leaky integrate-and-fire neuron. The variation of the inner potential $u_{post}^{I}\left(t\right)$, at time *t*, is obtained as follows:


(5) $$\begin{array}{r} \frac{\tau }{1.5}\Delta u_{post}^{I}=-u_{post}^{I}+\underset{pre}{\sum }\left(w_{post,pre}^{PI}\cdot a_{pre}^{P}\left(t\right)\right) \end{array}$$

where $a_{pre}^{P}\left(t\right)$ is the activation of a pre-synaptic CA1 pyramidal neuron, and $w_{post,pre}^{PI}$ is the connection weight between the pre-synaptic pyramidal neuron and the post-synaptic interneuron.

The neurons of the Subiculum have the following activation dynamics (without losing generality we assume a binary activation):


(6) $$a^{S}=\left\{ \begin{array}{l} 0\text{ for }{\text{a}}^{\text{P}}<\theta^{\text{+}}\\ 1\text{ for }{\text{a}}^{\text{P}}>\text{=}\theta^{\text{+}} \end{array}\right.$$

This function ensures that the subiculum neurons activate only when the corresponding neurons activate above a certain threshold θ^+^ (Supplementary Table 3) thus realizing a familiarity detection mechanism applied to the input pattern (see Section 2.1).

The ACC/PAL neuron (that has an inhibitory connection toward the VTA dopaminergic neuron) has the following activation dynamics (without losing generality we assume a binary activation):


(7) $$a^{AP}=\left\{ \begin{array}{l} 0\text{ for}\sum {\text{a}}^{\text{S}}\;\text{= 0}\\ 1\text{ for}\sum {\text{a}}^{\text{S}}>\text{0} \end{array}\right.$$

The VTA dopaminergic neuron has the following activation dynamics (again, without losing generality, we assume a binary activation):


(8) $$\text{DA = }\left\{ \begin{array}{l} 1\text{ for }{\text{a}}^{\text{AP}}\text{= 0}\\ 0\text{ for }{\text{a}}^{\text{AP}}>\text{0} \end{array}\right.$$

The dopaminergic neuron is active with 1 when it is not inhibited by the ACC/PAL neuron, and with 0 when the ACC/PAL neuron activates, thus implementing a dopamine modulation mechanism by novelty detection.

### 2.4. Synaptic plasticity

The plasticity of CA1 afferent excitatory connections from the input layer is based on a Hebbian-like rule with homeostasis, in order to implement both potentiation and depression, that prevents unlimited connection weight growth. In our model, the input signal is continuously sent to each pyramidal neuron in the network and therefore each individual neuron has to detect by itself when the input changes. For this purpose, the learning rule takes into account the local variation over time of the pre-synaptic signal and the variation of the post-synaptic potential of the CA1 pyramidal neuron. Furthermore, the learning rule also avoids learning during the initial transient time when a new signal is received, but the activation of the pyramidal neuron in CA1 may still not be related to the input signal. The learning rule also takes into account the activation level of the CA1 pyramidal neuron to avoid updating synaptic weights when its activation is too low. This prevents the update of synaptic weights when the input signal is absent or too low to be distinguished from noise.

As a consequence of the architecture containing lateral connections, CA1 tends to have a hill-shaped activation of the winning population. The synaptic weights are thus updated in proportion to such activation and so tend to further strengthen and refine the hill-shaped CA1 activation in correspondence to familiar patterns. However, learning stops when the activation of at least one CA1 pyramidal neuron reaches a sufficiently high level (a little below its saturation level) above the subiculum activation threshold, to avoid that learning tends to progressively recruit an unbounded number of CA1 neurons located around the peak neuron.

The excitatory synaptic weight of a pyramidal neuron $w_{post,pre}^{INP}$ is updated with the following equation, where $\Delta w_{post,pre}^{INP}$ denotes its variation:


(9) $$\begin{array}{r} \Delta w_{post,pre}^{INP}=HF\cdot LT\cdot SA \end{array}$$

The components of the Equation (9) have the following definition and meaning:

$HF=DA\cdot a_{post}^{P}\cdot \left(a_{pre}^{INP}-w_{post,pre}^{INP}\right)$, represents the Hebbian Factor, where *DA* is the dopamine (*DA*=1 when phasic dopamine is present, and *DA*=0 otherwise) and $a_{post}^{P}$ is the activation of the post-synaptic CA1 pyramidal neuron; this term is a variation of Hebb's principle inspired by Oja's rule (Oja, 1982) and modulated by dopamine (Gerstner et al., 2018); this rule updates the synaptic connections of highly active (“winning”) neurons and protects the connections of all other neurons; the dependence of this Hebbian factor from DA is the first of the two key mechanisms of the model that prevent catastrophic forgetting;

$LT=a_{post}^{P}>\theta^{-}$, represents the learning threshold factor, where θ^−^ is the threshold parameter defined in Supplementary Table 4; this term prevents learning when the activation of a CA1 pyramidal neuron is very low and thus avoids interference due to noise;

$SA=\left({ȧ}_{post}^{P}\left(\delta_{1}\cdot \tau \right)>\delta_{2}\right)\left({İ}_{post}<0.012\right)$, represents the stable activation factor, where ${ȧ}_{post}^{P}\left(\delta_{1}\cdot \tau \right)$ is the time derivative of $a_{post}^{P}$ computed as deviation from the moving average over a time interval δ_1_·τ; the values of δ_1_ and δ_2_ are defined in Supplementary Table 4; this term represents a condition that allows learning only in presence of stable activation, and prevents the model from learning in presence of noise or residual activation due to the previous image; the parameter 0.012 was manually tuned to trigger learning only when the İ_*post*_ is sufficiently small (the input signal is perceived as sufficiently stable).

Learning also affects the lateral inhibitory connections of CA1. This is the second one of the two key mechanisms that prevent catastrophic forgetting in the model and is inspired by the work of Udakis et al. (2020). To avoid the dominance of the CA1 pyramidal neurons that have already learned an image, the model increases the inhibition of those neurons while they are learning. Importantly, Udakis et al. (2020) showed two different learning rules for parvalbumin and somatostatin interneurons. In our model, interneurons provide lateral inhibition between excitatory CA1 neurons, but not feedforward inhibition from the Schaffer pathway. We thus considered them as somatostatin interneurons (Stefanelli et al., 2016) and implemented a plasticity rule compatible with the findings of Udakis et al. (2020).

For this purpose, the inhibitory synaptic weight $w_{post,pre}^{IP}$ is modified during the learning process with the following plasticity rule, where $\Delta w_{post,pre}^{IP}$ is its variation:


(10) $$\begin{array}{r} \Delta w_{post,pre}^{IP}=LF\cdot LT\cdot SA\cdot ELL \end{array}$$

The components of the Equation (10) have the following definition and meaning:

$LF=-\frac{L}{\tau }\cdot DA\cdot a_{post}^{P}\cdot \left(a_{pre}^{I}+0.3\cdot w_{post,pre}^{IP}\right)$ represents the learning factor for inhibitory synapses, where *L* is a constant parameter defined in Supplementary Table 4. In presence of dopamine, plasticity is proportional to the neuron activation level: this strengthens the inhibitory connections of highly active (“winning”) CA1 neurons while affecting fewer other neurons.

*LT* and *SA*, respectively represent the learning threshold factor and the stable activation factor and they are the same as those in Equation (9);

$ELL=\frac{\overset{N}{\underset{pre}{\sum }}|a_{pre}^{INP}-w_{post,pre}^{INP}|}{N}>0.004$ represents the excitatory learning level, where *N* is the number of input neurons (Supplementary Table 2), which is equal to the number of input synapses of one CA1 pyramidal neuron; this term is inversely proportional to the learning level of the excitatory synapses (the value of this term decreases as the pyramidal neuron learns) and in this way it reduces the plasticity of the inhibitory synapses while the neuron learns; the parameter 0.004 was manually tuned to trigger learning only when the excitatory synaptic weight update is sufficiently large as this signals that the neuron still needs to learn.

### 2.5. Experimental setup and tests

We tested the model with two different image datasets and with two different experimental procedures. We introduce here the rationale for using such datasets and experimental procedures and then further specify their features in the subsections below.

The first dataset is the classic machine-learning dataset MNIST (LeCun et al., 1998, 2010; LeCun, 2013), and the second consists of images taken from a landscape of the city of Lake Como. The purpose of using two image datasets having different features was to test the behavior of the model and in particular its two core mechanisms used to face catastrophic forgetting. The MNIST dataset, a standard machine-learning dataset, consists of handwritten numerical digits, while the images of the Lake Como landscape consist of nature photos taken from a real scenario. The different source of these images implies different characteristics of contrast and brightness and also different content and amount of details. All original images were converted to grayscale images with 256 levels of gray, and a resolution of 40 ×40 (1600) pixels corresponding to the size of the input layer (Figure 1). The two sets of images can be approached with similar model parameters, as shown in the Supplementary Table 4. The parameters that were different in the two experiments were related to the different brightness and contrast of the images of the two sets. In real organisms, these differences would be compensated for by appropriate adjustments of the eye and early visual areas that are not represented in the model (Rahimi-Nasrabadi et al., 2021).

**Figure 2:**
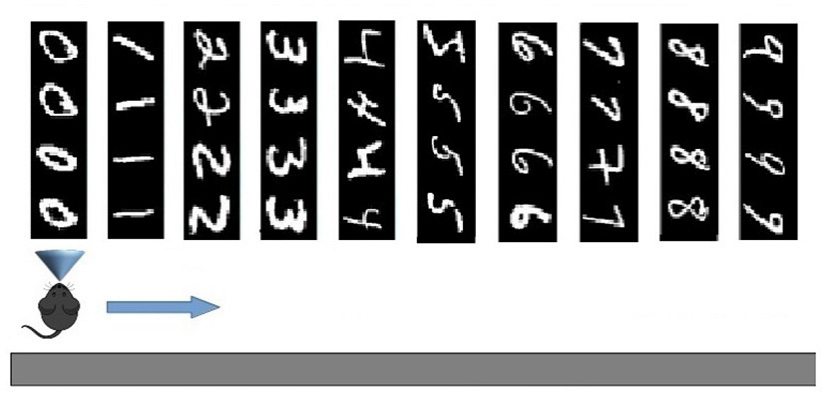
Test on Catastrophic forgetting experiment. The system learns each of the 10 tasks (each of which represents a numerical digit) by looking at its constituent images once. Then the system is tested with the repetition of all tasks, while plasticity is turned off, to evaluate catastrophic forgetting. The number of images correctly classified for each task provides a measure of the level of recognition of the task. The test is repeated several times by changing the learning order of the tasks, and, on this basis, the average level of catastrophic forgetting per task is calculated.

The two experimental protocols were directed to test different aspects of learning interference. In particular, the catastrophic forgetting experiment follows the classic protocol (French, 1999; Allred and Roy, 2020) in which an agent learns a large number of blocks of class-specific stimuli one after the other (in our setup 10 blocks of 4 images each, see Figure 2). This allows measuring the degree of interference in terms of the capacity of correct classification of a block class when the number of blocks learned after it progressively increases. The key of the test is hence the possibility of measuring the “speed” of forgetting when new stimuli blocks are progressively learned. The experiment, therefore, focuses on the study of catastrophic forgetting as a function of progressive learning over time. In addition, the experiment also allowed us to test the ability to generalize within each class block.

**Figure 3:**
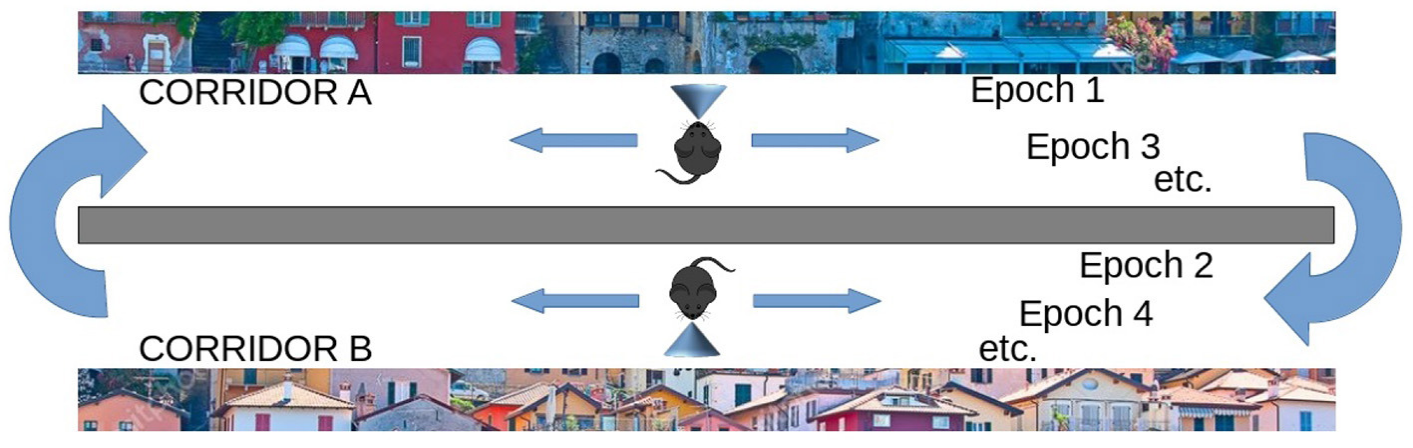
Life-long learning experiment with images of Lake Como. The agent moves down the first corridor seeing and learning each image observed in each of the next 20 locations and repeats the corridor until all images have been fully learned (Epoch 1), then the agent moves to the second corridor and repeats the process until all images in the second corridor have been fully learned (Epoch 2). Then the agent goes back to visit the first corridor (Epoch 3) and learns only the unrecognized images. The agent then moves to the second corridor and does the same. The process is repeated by alternating between the two corridors until all the images in both corridors have been fully learned and recognized. The experiment allows evaluating whether and to what extent the representations of the different images by the different neural groups encoding them are stable when the agent has other experiences.

On the other hand, the life-long learning experiment is more ecological in the sense that it does not involve classification tasks, but the recognition of two different tasks after their free learning, with the aim of observing how the respective population codes, represented by the position within the network of the peak neuron (the most excited of a population), change or stabilize as the agent moves from one task to another. This experiment type was inspired by the experiment of Udakis et al. (2020). Each task requires the model to decide by itself when it has been fully learned before moving on to the next one; this may involve looking several times at the images that make up a single task. In particular, the test is directed to test the amount of the “shift” of several internal representations (place cells), related to well-learned stimuli taken from a certain environment (in our case the images taken from a corridor, see Figure 3), when another large set of stimuli from a new environment are learned in depth (images taken from a second corridor). The “shift” is measured by evaluating if the neurons that encoded the images of the first environment change their representations after learning the images of the second environment. The experiment thus focuses on studying catastrophic forgetting in (neural) space. This test is relevant as Udakis et al. (2020), proposed it to study the stability of receptive fields of place cells based on the plasticity of lateral inhibitory connections.

#### 2.5.1. The MNIST handwritten digit dataset

In this setup, the network model was tested using the MNIST images. Every single image observed by the network (field of view) had a size of 40 ×40 pixels. In particular, we used the first four images for each digit (0–9), or class, of the dataset, thus having a sequence of 40 images grouped into 10 consecutive subsets, or learning tasks. Each task consisting in learning 4 images corresponds to learning one of the digits from 0 to 9 (Figure 2). In addition to such 40 images, we also used other 40 images (4 from each digit) as a test-set to perform a generalization test.

#### 2.5.2. Dataset of naturalistic images of the city of Lake Como

The naturalistic images are taken from the landscape of the city of Lake Como. The images were divided into two sets representing two distinct landscapes, each composed of 20 contiguous images, for a total of 40 images. Every single image observed by the network had the size of 40 ×40 pixels. The two landscapes contain images with different brightness and contrast and are very rich in detail.

#### 2.5.3. Catastrophic forgetting test: Experiment procedure

In this experiment, the model has to perform 10 learning tasks, each of which involves observing four consecutive images only once. After the learning phase, all images are presented to the model in the same order, with plasticity turned off, to check which ones are recognized. The logic of the test is that the more the model is affected by catastrophic interference, the more it will tend to not recognize the images observed and learned at the beginning of the sequence. The experiment was repeated with both the MNIST and the naturalistic images to study the effects of different features of the two datasets.

When using the MNIST image dataset, the four images that make up a learning task all correspond to the same class, one of the digits 0–9. To measure whether an image was recognized, we followed this procedure: (a) during learning, each pyramidal neuron (in CA1) whose synaptic weights were modified by the learning rule of the Equation (9) was labeled with the digit class corresponding to the learned image (an example is shown in **Figure 7**); (b) during the test, an image was considered correctly recognized if the neuron that fired the most was labeled, during learning, with the same digit class as the subset to which the current image belongs. Note that because each subset was formed by 4 images, the average recognition rate of a subset could have one out of five possible values: 0, 25, 50, 75, or 100%.

For the experimentation with naturalistic images of the Lake Como landscape, we used a similar procedure with the following differences. As with MINST, we considered 10 subsets of 4 images each, but this time these subsets did not correspond to any class, so instead of the class number, we used a number from 0 to 9 as a label, which uniquely identified the subset. Note that in each MNIST subset, the 4 images in it refer to the same class of digits and are therefore more similar to each other than those in other subsets that refer to other classes of digits. In contrast, in the naturalistic dataset, all images within and between different subsets have a comparable degree of similarity. This allowed us to test the robustness of the system by grouping images based on the task to be performed and not based on the greater similarity between the images.

Also in this case the experiment consisted of learning, one after the other, 10 sequences of 4 images each, and then measuring the recognition percentage of each sequence, while the learning was disabled. Specifically, the following steps were implemented: (a) during learning, each neuron whose synaptic weights were changed according to the learning rule of the Equation (9) was labeled with the number identifying the subset containing the learned image; (b) during the test, an image was considered correctly recognized if the neuron that fired the most was previously labeled with the same number as the subset of the current image. Again, since each subset consisted of 4 images, subset recognition could have only five values: 0, 25, 50, 75, 100%.

In the case of MNIST images, we also tested the generalization ability of the model in correctly classifying digits even in the presence of images other than those used for learning. This was possible by using different images for testing than those used for learning since the MNIST dataset contains many different images that refer to the same digits.

Since the results could be influenced by the specific characteristics of the images that form the sequence of tasks, we repeated the experiment 10 times mixing each time the order in which the 10 tasks were learned. In particular, we changed the learning order of the 10 tasks by applying permutations to the initial order: the result reported represents the average of the results over 10 permutations.

The experiments described above were performed with both the wholly functioning model and the model in which we deactivated the dopamine modulation (this was done by preventing the dopamine stopping mechanism) and the plasticity of inhibition (this was done by preventing the learning process). Under these conditions dopamine release does not stop when the novelty disappears, so the learning of an image would never stop if left under the control of dopamine. Therefore, after a maximum time has elapsed, the model is always forced to move on to observe the next image.

#### 2.5.4. Lifelong learning test: Experiment procedure

The second class of experiments simulated an agent (e.g., a mouse) that moves along two virtual corridors. As it moves through each corridor, the agent stops at 20 successive positions and observes the resulting landscape image at each position (Figure 3). In each position, the agent can learn the observed image and then move on to the next when the dopaminergic neuron is inhibited, thus indicating that the image has been learned. Note how this implies that the agent autonomously decides when to pass to the next position. However, after a maximum observation time has elapsed, the model is always forced to move on to the observation of the next image. The visit of the entire corridor is repeated several times until all images are fully recognized, that is, until there is no more novelty signal, and dopamine stops being released during the exploration of the entire corridor. Note that, in a subsequent passage in the corridor, it may happen that an image already observed previously may not be recognized when it is observed again, for two reasons: either because the image was not fully learned during the previous visit, or because the learning of subsequent images interfered with its encoding. The whole test ends when the agent crosses the two corridors recognizing all the images observed, without producing any signal of novelty.

The aim of these experiments was to evaluate the efficiency of the model during a long learning process, alternating two different environments (corridors), and observing how the respective population codes, represented by the position within the network of the peak neuron (the most excited of a population), change or stabilize as they move from one corridor to another. Unlike the previous catastrophic interference test, here the agent had the opportunity to relearn the non entirely familiar images until they become fully recognized before passing to the next environment (corridor).

In these experiments, we define “epoch” the time taken by the model to fully learn a set of 20 images corresponding to a single virtual corridor. Each time the agent changes the corridor, we increase the total number of epochs by 1. We also define a “learning episode” the learning of a single image. To measure learning efficiency we counted the number of learning episodes for each epoch, and the number of epochs needed to learn the images of both corridors. Figure 3 illustrates the process.

The Lifelong learning test was also repeated with a model in which we deactivated dopamine modulation and/or plasticity of inhibitory connections, following a procedure similar to the one described above for the case of normal functioning. In the condition in which the absence of dopaminergic modulation prevents the agent from autonomously switching to the next image, in order to prevent it from remaining in observation of the same image forever, the agent is forced to switch to the next image after a predefined maximum time.

## 3. Results

### 3.1. Tests on catastrophic forgetting with the MNIST dataset and naturalistic images of Lake Como

**Figure 4:**
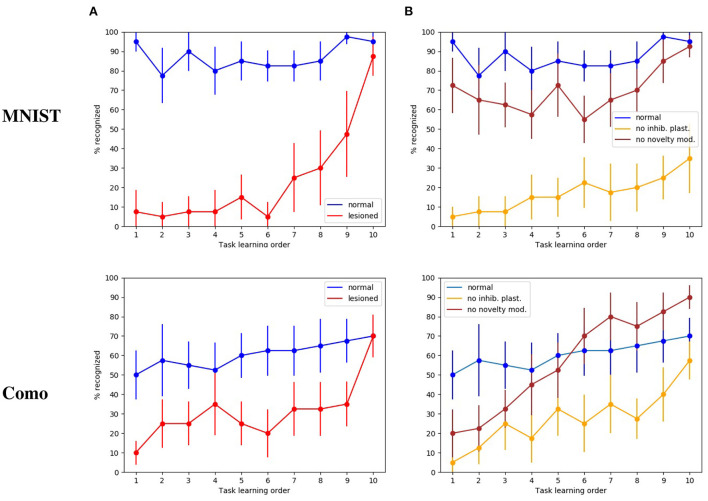
Test on catastrophic interference with MNIST and Lake Como images. **(A)** Comparison between the normal functioning model and the lesioned model (without dopaminergic modulation and without plasticity of inhibitory synapses). **(B)** Normal functioning is compared with the two conditions where only inhibitory synapses plasticity or only dopaminergic modulation is deactivated. The data were collected after training the model by performing 10 tasks in succession, each of which consisted of learning 4 images. The graphs show the mean and standard deviation over 10 repetitions of the test, performed by changing the order of the tasks through 10 permutations of the initial task order.

The first tests were directed to evaluate how the whole intact model behaves with respect to catastrophic interference. Figure 4A shows that in this condition the model has a high recognition rate with the MNIST images, in particular between 80 and 90%, and close to 100% for the last subset. Instead, with the naturalistic images (Lake Como images) the recognition rate gracefully degrades from about 70% with the recently experienced images (subsets 9 and 10) to about 50% with the first experienced images (subsets from 1 through 4).

The next test was directed to study the effects of catastrophic interference when the novelty detection mechanisms is deactivated (that is when dopamine was always present), and the inhibitory plasticity was absent. With the MINST images, the quality of memory drops drastically, from about 90% for the last learned image subset to about 10% for the image subsets learned at the beginning of the sequence (Figure 4A). With the Lake Como images, the quality of memory also drops drastically, from about 70% for the last learned image subset to about 30−10% for the image subsets learned at the beginning of the sequence (Figure 4A). This demonstrates the effectiveness of the combination of the two mechanisms in the protection from catastrophic interference.

The differences in the recognition rate of the two datasets can be explained by the experimental procedure of this experiment. Indeed, the procedure consists in learning ten groups of four images and requires recognizing the *class* of each image. This tends to increase performance with the MNIST dataset since each subset refers to the same numerical digit and contains images very similar to each other. Instead, the Lake Como images belonging to each subset are different from each other and this makes the task more difficult.

In order to understand the relative contribution of dopamine regulation and of lateral inhibitory plasticity, we analyzed the catastrophic interference by deactivating the two mechanisms separately. Figure 4B shows that in both cases a catastrophic interference effect occurs. However, the lesion of the novelty mechanism results in a milder impairment compared to the blockage of the inhibitory plasticity. Again, we observed that, when the model is tested with the MNIST dataset, the performance is higher than with the Como dataset. Moreover, there is protection from catastrophic interference if the lesion only involves the novelty mechanism, while the lateral inhibition is still in place (Figure 4B). In the case of MNIST without novelty-induced modulation of dopamine, we see that catastrophic interference is milder than for the Como images. The reason for this effect is due to the easier generalization within the same subgroup of MNIST digits, which counterbalances the blockage of novelty. Instead, the Como images are different even within the same class, so this generalization effect is not present. In the case of Como images, the performance of the model in Task 10 is higher when there is no dopamine modulation by novelty, with respect to the wholly functioning model. The reason is that the learning process never stops and, consequently, the last images are overtrained. This phenomenon is not visible with MNIST images because in this case the wholly functioning model already reaches a maximum level of performance on the last tasks.

**Figure 5:**
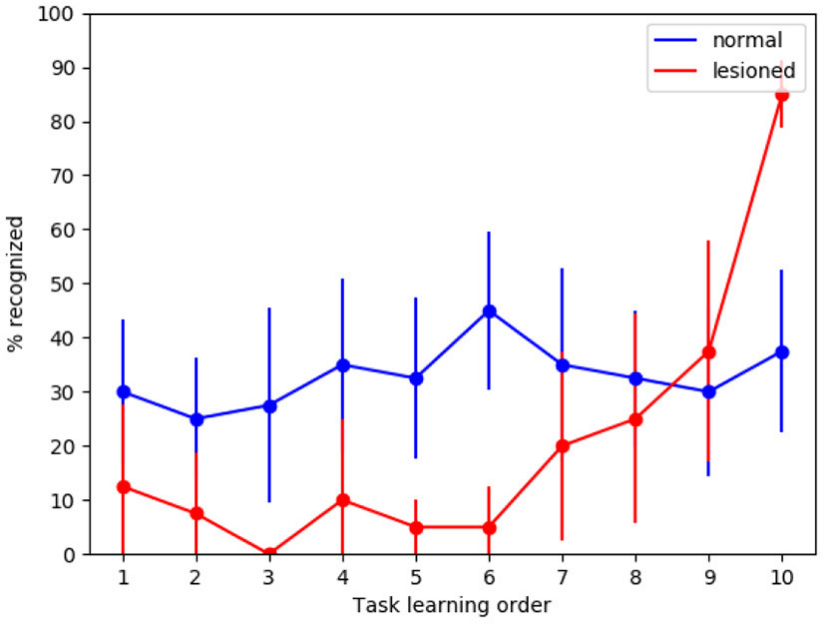
Tests of generalization with the MNIST dataset using a different test dataset, run with the wholly functioning model and the lesioned model (without dopamine modulation and without inhibitory plasticity). The values reported in the graphs are averaged over repeated tests each of which was performed by changing the order of execution of the tasks (using 10 permutations of the initial task order). The vertical bars represent the standard deviations.

To investigate if the model is indeed capable of generalization, we tested it with new MNIST images, that is, using for the test an image set which is different from that used for training. We can see in Figure 5 that in the case of normal functioning the digits of the test set are recognized with an accuracy of about 35%. Notwithstanding performance does not achieve high levels, this is an interesting result considering that the system has learned to classify never-seen-before MNIST images by looking at only 4 images of each class once.

As expected, in the case of the lesioned model the result shows catastrophic interference. Again, the removal of dopamine modulation by novelty causes learning to never stop. This produces the effect that system performance for task 10 is higher in the lesioned model than in the fully functional model.

#### 3.1.1. The memory content of the network

**Figure 6:**
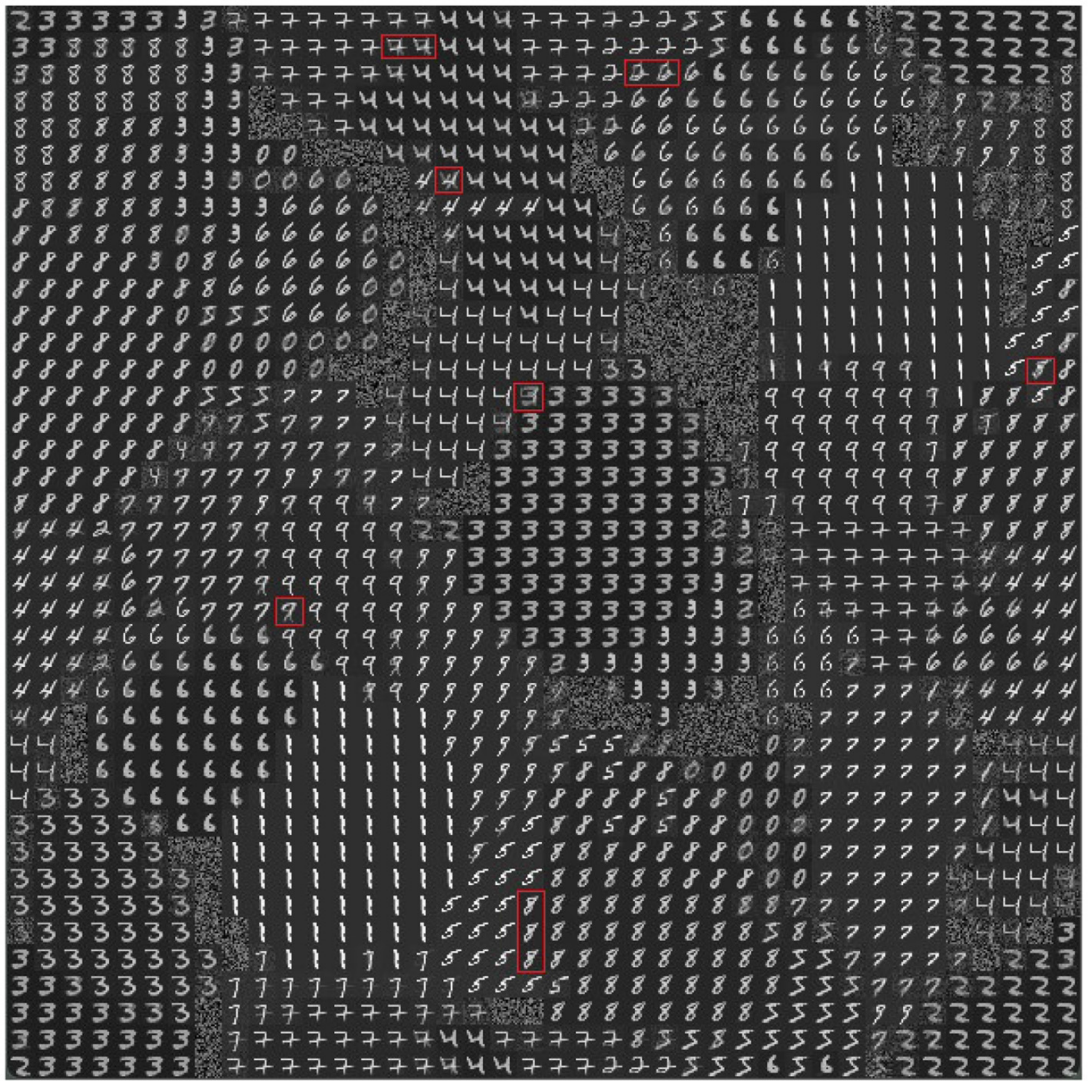
Representation of the model memory content after learning some images of the MNIST dataset (10 MNIST digits, 4 images per digit, for a total of 40 images). Each small image (digit or blurry image) in the 40 ×40 cells of the graph consists of 40 ×40 pixels representing the incoming connection weights of the excitatory synapses of the pyramidal neuron at the corresponding position in the 2D neural map representing the pyramidal layer of CA1. White represents the highest value of the weights and black represents the lowest value. The red rectangles highlight some of the neurons, located near the boundary of adjacent populations, that encode a mix of digits.

**Figure 7:**
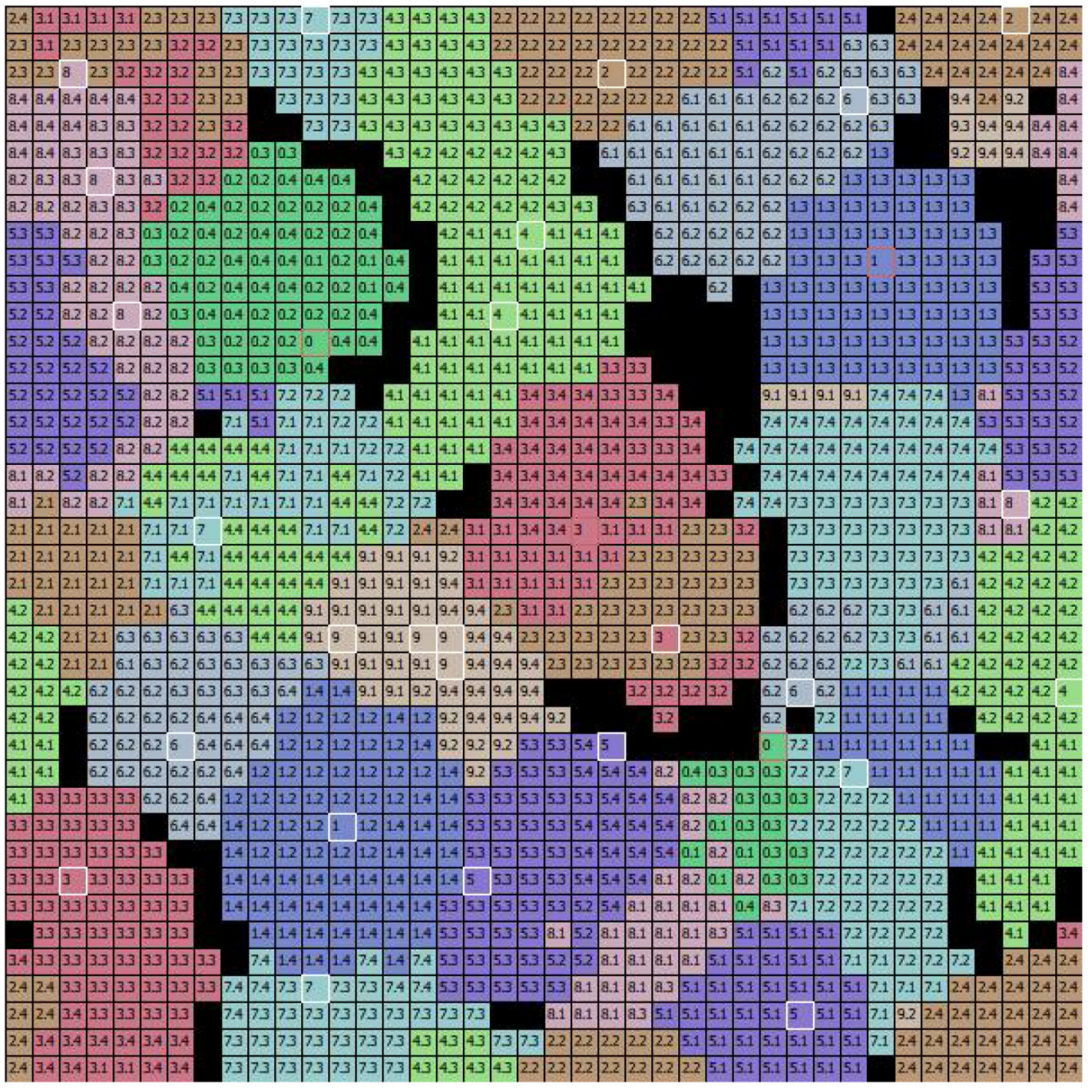
The specialization of excitatory CA1 pyramidal neurons after learning the MNIST image dataset. Each two-digit number in the cells of the graph indicates the image learned by the neuron located at that position within the 2D map of CA1: the first digit of the number in the cell indicates the class, and the second digit after the dot indicates the image position within the sequence (of the 4 images that belong to the class). During the learning phase, the class and the number of the image were assigned to each “winning” neuron when its synaptic weights were updated. Different colors represent different populations, each of which has learned a specific class of digits. The cells highlighted in white identify the peak neuron for each population responding to one single image, and the cells highlighted in red identify the peak neuron responding to two images of the same class.

To investigate the internal organization of the model that led to the results illustrated above, we evaluated the memory content that the model acquired with learning. Figure 6 shows the weights of the connections linking the input excitatory neurons to the CA1 pyramidal neurons after learning the 10 MNIST digits (40 MNIST different images). In Figure 7, where different colors represent different populations, it can be seen that the population code for each learned image is composed of a series of neurons that have undergone changes in their excitatory synaptic weights to varying degrees.

To understand the responsiveness of individual neurons, we compared the synaptic weights, shown in Figure 6, with the MNIST digit best recognized by each neuron. For this purpose, Figure 7 shows the images classified by the CA1 pyramidal neuron populations after learning the MINST images. Comparing Figure 6 with Figure 7, it can be observed that neurons encoding images related to the same class tend to be close in the neural (physical) space. This is due to the local nature of the connectivity of the neurons, as shown by self-organizing maps (Kohonen, 1982; Hazan et al., 2018). The observation of the two figures suggests that the neurons that are at the boundaries between two neural areas encoding different classes of digits (Figure 7), tend to encode a mix of digits (Figure 6, highlighted by red rectangles). Figure 7 also shows that the learned images employed almost all the neural resources available in the network (black cells indicate neurons that were never active for any image during learning).

**Figure 8:**
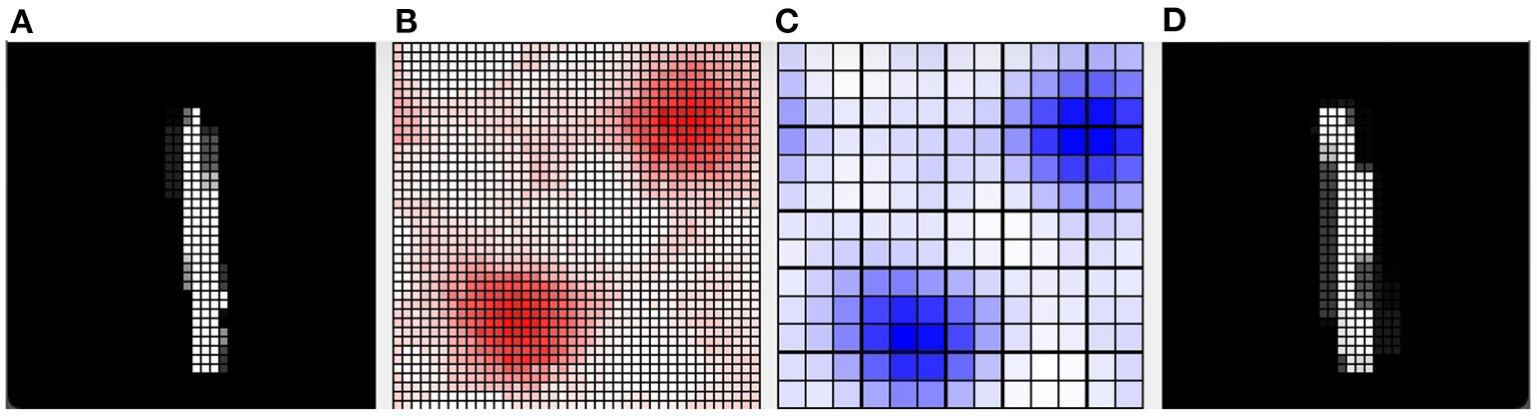
Example of how the CA1 neurons respond to a MNIST image after learning. **(A)** The input image representing the digit “1”; the gray scale of the pixels is proportional to the level of activation of the input layer ranging in [0, 1]. **(B)** Activity of all the excitatory neurons (in red) and **(C)** activity of all the inhibitory neurons (in blue) of the CA1 in response to the input image. Each cell of the graphs corresponds to a neuron, and the color intensity reflects its activation level ranging in [0, 1]. **(D)** The weights of the connections between the input and the excitatory synapses of the neuron that is the most active in response to the input image. The grayscale is proportional to the level of each weight ranging in [0, 1]. In this case, the network responded with the peak neuron that learned a slightly different image of the same class because it was not able to distinguish the differences.

Furthermore, we present in Figure 8 an example of the responsiveness of the network to an image of the digit class “1” after learning 4 other images of the same class. In Figure 8A, we can see the input image observed. Figure 8D shows the weights of the connections between the input and the pyramidal neuron that responded with the highest activation to the observed image. It can be seen that, although similar, the observed image and the representation of the weights of the neuron that is most responsive to it slightly different. This represents a case where the network was unable to distinguish the differences between the two images, but still correctly classified the input image as belonging to the digit class “1”. Figure 8 also shows the activation of neurons in the pyramidal layer (Figure 8B), and interneurons in the inhibitory layer (Figure 8C), in response to the observed image. The highest level of activation involves a small group of excitatory neurons and a corresponding small group of inhibitory neurons. On the other hand, the neurons that encode other images have a very low activity because the synaptic weights from the input are too small. In this way, they are protected from interference of the currently perceived image.

### 3.2. Experiments of life-long autonomous learning with Lake Como images

This group of experiments aims to evaluate the ability of the network to carry out a continuous unsupervised autonomous learning and recognition task of two long sequences of naturalistic images, representing two distinct landscapes of Lake Como city. The two sequences are learned several times, therefore the final interference level is reduced to a minimum. In this condition, the dynamics of the experiments highlight the possible shift of neural image representations within the CA1 neural map, which could resemble the place field shift observed in Udakis et al. (2020).

#### 3.2.1. Life-long learning in the wholly functioning model

**Figure 9:**
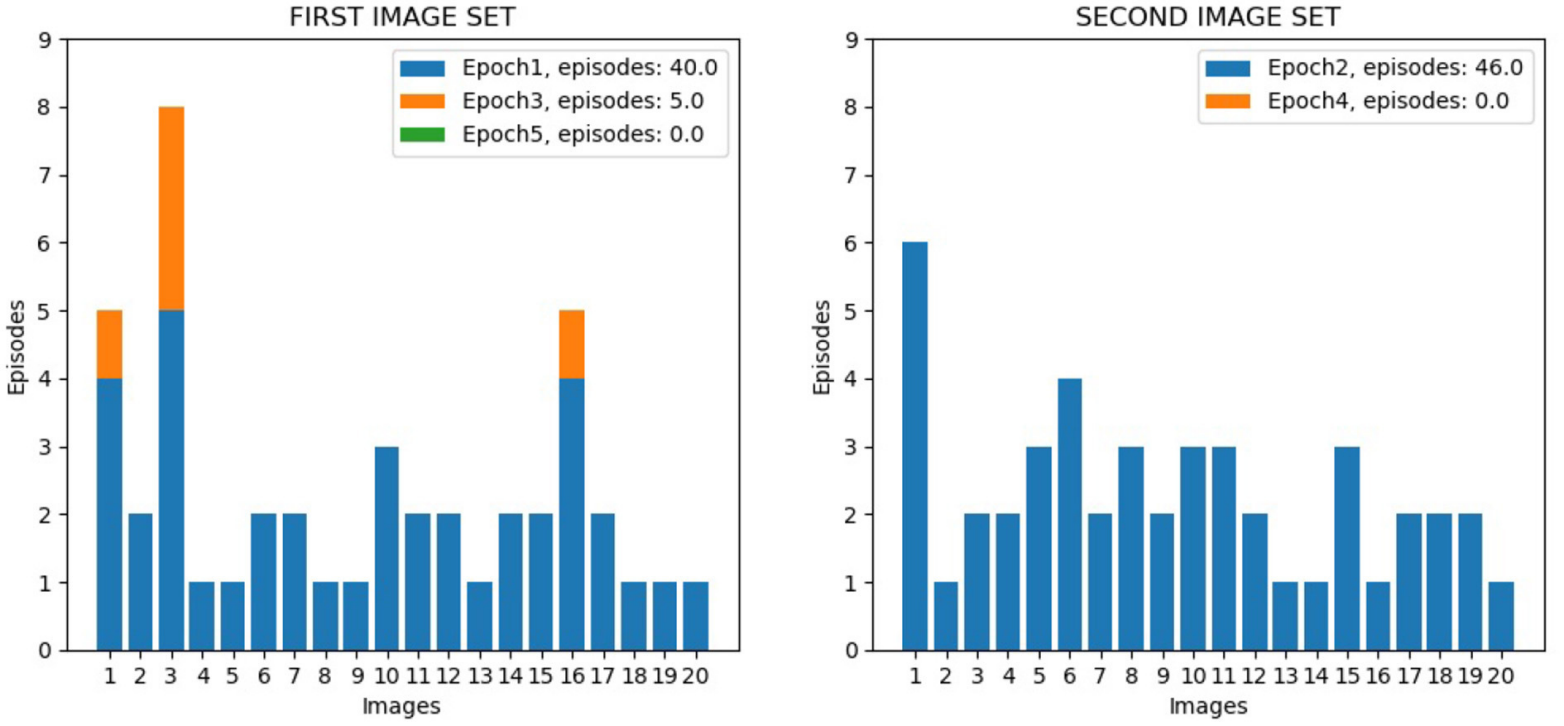
Life-long learning test with Lake Como images, model performance. The left graph shows the data for the 20 image sequence from the first corridor, while the right graph shows the data for the 20 image sequence from the second corridor. The graphs show the number of visits to the same corridor (learning episodes) to complete an epoch; the colors indicate the different epochs.

The first test was directed to investigate the overall performance of the wholly functioning model. Figure 9 shows the number of learning episodes for each image, using different colors for different epochs (each involving multiple visits to the same corridor until all its images are learned, Section 2.5.4). Within the same epoch, some images required more than one episode to be learned: (1) First epoch: the first corridor was explored for the first time, and all the 20 images were learnt with 40 learning episodes; (2) Second epoch: the second corridor was explored for the first time, and all the 20 images were learnt with 46 learning episodes; (3) Third epoch: the first corridor was explored again, and additional learning was required for only three images for a total of 5 learning episodes; (4) Fourth and subsequent epochs: the second corridor and the first corridor were explored again, and no additional learning was required; by alternating the exploration of both corridors, further learning was no longer necessary. We can see from the figure how different images required a different number of learning episodes to complete the learning. This is because some images have a higher level of similarity with other images, and so they require a longer time to learn in order to better distinguish them. For three images of the first corridor, the model also needed further learning (epoch 3) after completing the learning of the second corridor (epoch 2). This phenomenon possibly occurred because these images are less distinctive than others and so are more sensitive to catastrophic interference.

**Figure 10:**
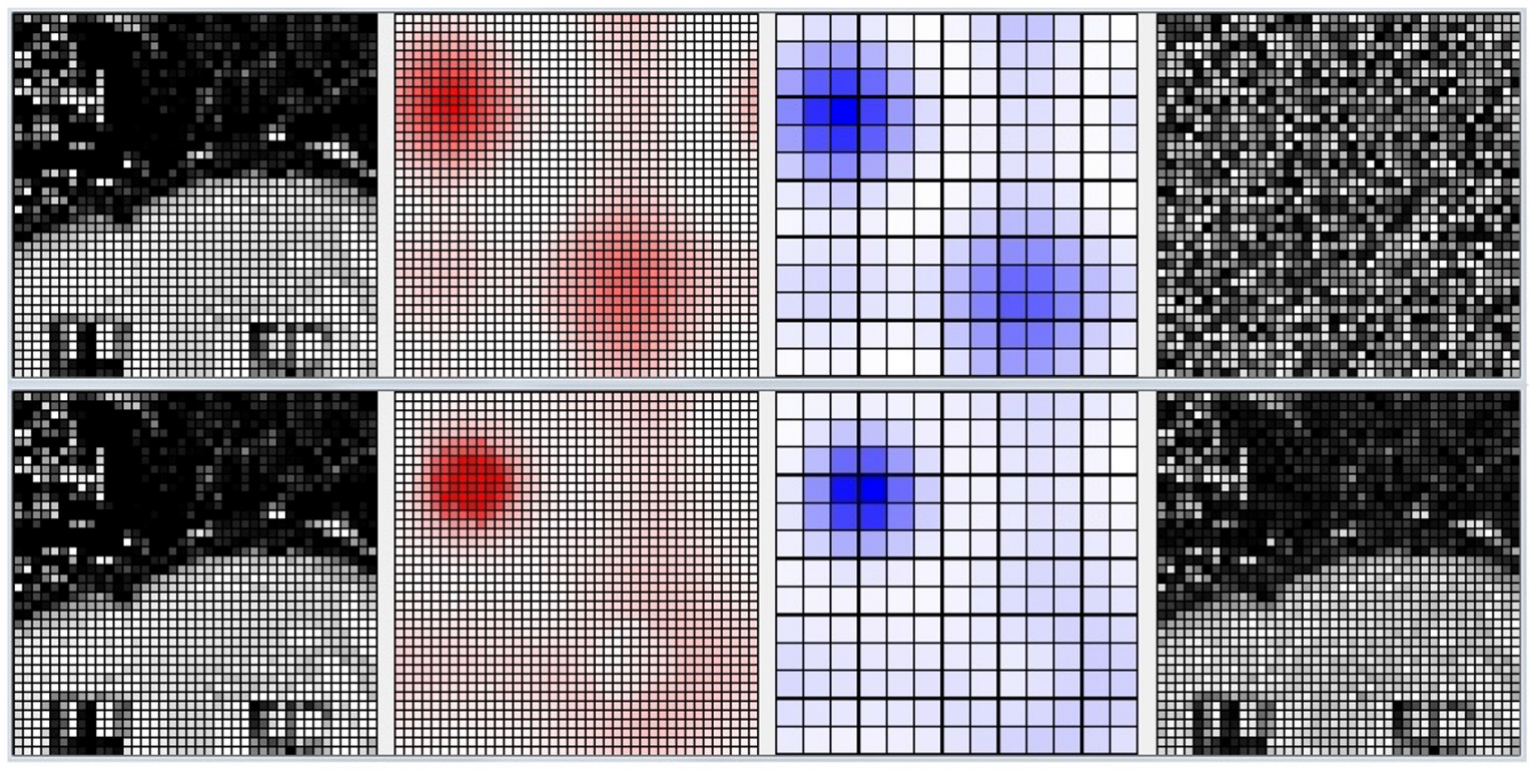
Life-long learning task, an example of the model CA1 activation with a Lake Como image from the second corridor. **Top**: before learning. **Bottom**: after learning. Picture from left to right: the image observed by the network; the corresponding activation level of the excitatory layer (darker red indicates higher activation); the corresponding activation level of the inhibitory interneuron layer (darker blue indicates higher activation); the synaptic weights of the connections from the input to the most active excitatory neuron in CA1.

Figure 10 shows the activation of the CA1 neurons corresponding to a Lake Como image from the second corridor, before learning (top) and after learning (bottom). When a new image is presented, the model reacts by activating a pattern of neural populations. This produces a novelty signal that triggers the learning of the perceived image. During the learning process, the excitatory and inhibitory synaptic plasticity of the most active excitatory neurons causes their specialization, and the dynamics of the network change until a new stable condition is reached in which a new population with a different shape emerges. At this point, the novelty signal ceases and the network stops learning because dopamine release stops.

#### 3.2.2. Life-long learning in the lesioned model

Next, we analyzed the role played by catastrophic-interference protection mechanisms in the life-long learning experiment. For this purpose, we performed the life-long learning experiment with Como images under three different conditions: with a wholly functioning model; with the model in which dopamine modulation and inhibitory plasticity were blocked; with the model without dopamine (thus without excitatory and inhibitory learning).

**Figure 11:**
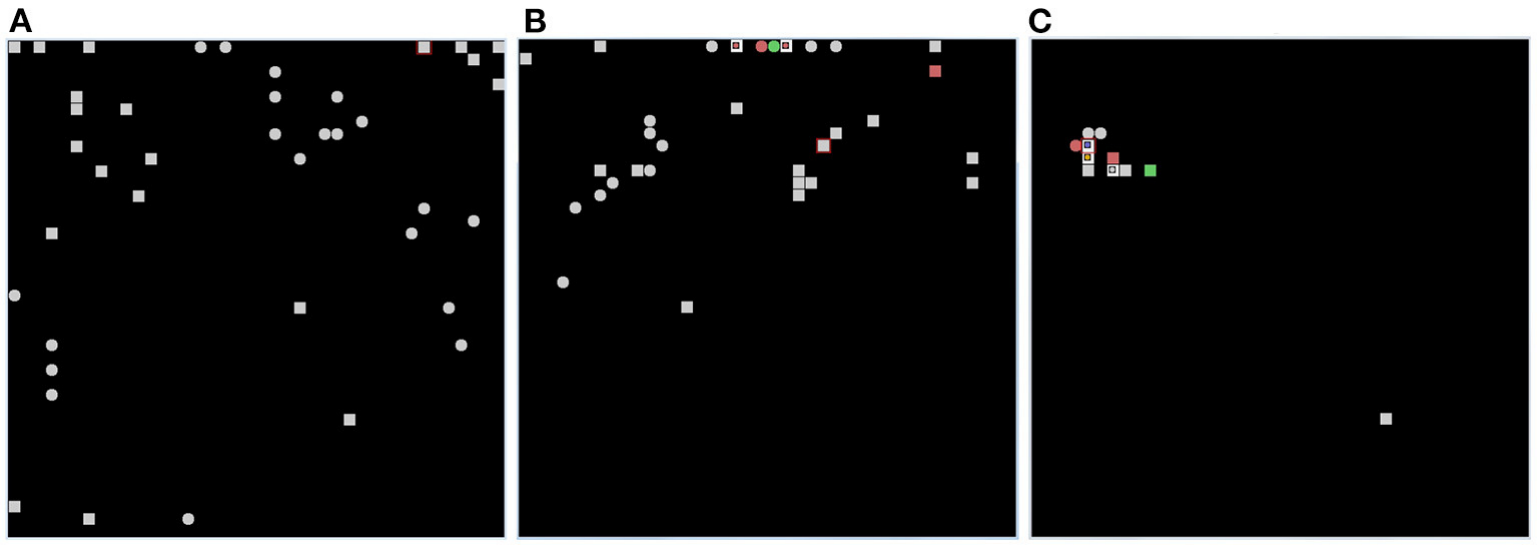
Results of the life-long learning task with Lake Como images. **(A)** With normal model operation. **(B)** Without dopamine modulation and without inhibitory plasticity. **(C)** Without dopamine release, there is no learning (both no excitatory and inhibitory plasticity), and the position of the neurons on the map is determined only by random synaptic weights that cause those neurons to have greater activation than others. Each of the three graphs was drawn after 4 epochs, in which in the case of normal operation **(A)** the map is stable and no longer changing, while in the case when both mechanisms are deactivated **(B)** the map is not stable but always changing. The symbols in each graph indicate the positions of pyramidal neurons, within the CA1 neural map, that are maximally activated for each of the 20 images in the first corridor (squares) and for each of the 20 images in the second corridor (circles). The red color indicates two images encoded by the same neuron. The green color indicates three images encoded by the same neuron.

Figure 11 shows that, after learning, the wholly functioning model forms distinct winning neurons/populations for the different images of the two corridors. In contrast, the lesion-affected model often uses the same neurons/populations to represent different images. The map is not stable because the model continues to relearn the images already learned using different populations. This shows that, without the two protective mechanisms, the model is affected by catastrophic forgetting. In the absence of learning all images are encoded by a few neighboring neurons in the neural (physical) space. These neurons correspond to those that are most activated on the basis of the random input connection weights. This happens because random weights could promote the activation of neurons in certain areas of the neural (physical) space, and all the images presented are more similar to each other than to the uncorrelated random weights. Moreover, once a population is maximally active for one image, it tends to inhibit other populations and thus win for subsequent images as well. This effect is due to the fact that, in order to have greater biological plausibility, the system is not reset between the observation of different images as is the case in other models (Section 2.1).

To check the stability of these results, we repeated the experiment three times for each operating condition, with three different values of the random generator seed used to set the initial values of the synaptic weights. In Supplementary Table 5, we report the average and standard deviation of the number of distinct neurons having maximum activation caused by all experienced images. A number similar to that of the images to encode (40) means that the model represented them in distinct neural groups; instead, a lower number means that the model encoded some images with the same neurons. These data confirm the results that the two mechanisms are very effective in protecting acquired representations from interference.

#### 3.2.3. Representations of instability caused by catastrophic forgetting in life-long learning tasks

Given that novelty-induced dopamine modulation and inhibitory plasticity are jointly important in lifelong learning settings, we investigated their relative contribution. For this purpose, we used Lake Como and MINST datasets to better understand the effects of the two mechanisms depending on the different properties of the two image datasets.

**Figure 12:**
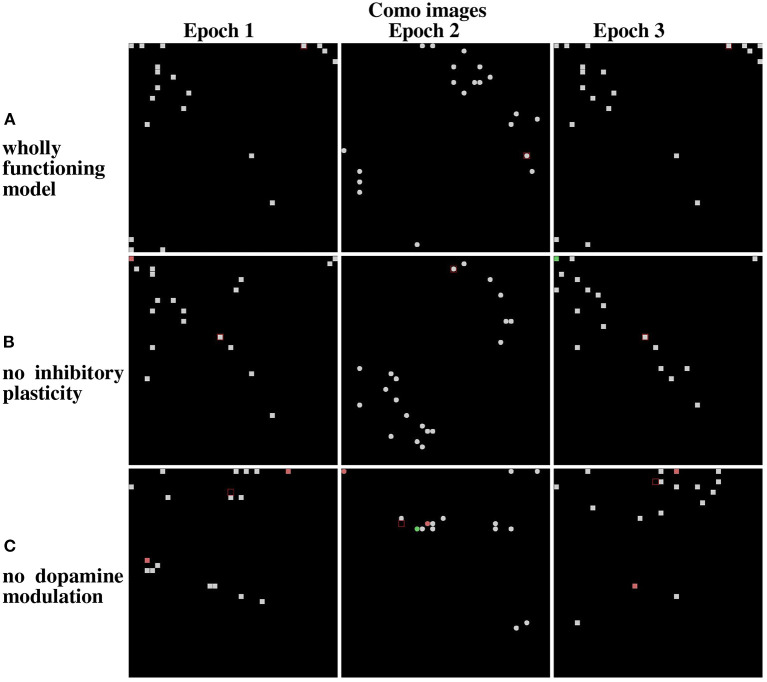
Life-long learning test: peak firing neurons corresponding to Lake Como images. **(A)** Normal model operation. **(B)** Model working only with dopamine modulation (no inhibition plasticity). **(C)** Model working only with inhibition plasticity (no dopamine modulation). The maps on the left show the peak firing neurons after the first pass of the first corridor (Epoch 1). The maps at the center show the peak firing neurons after the first pass of the second corridor (Epoch 2). The maps on the right show the peak firing neurons after the second pass of the first corridor (Epoch 3).

**Figure 13:**
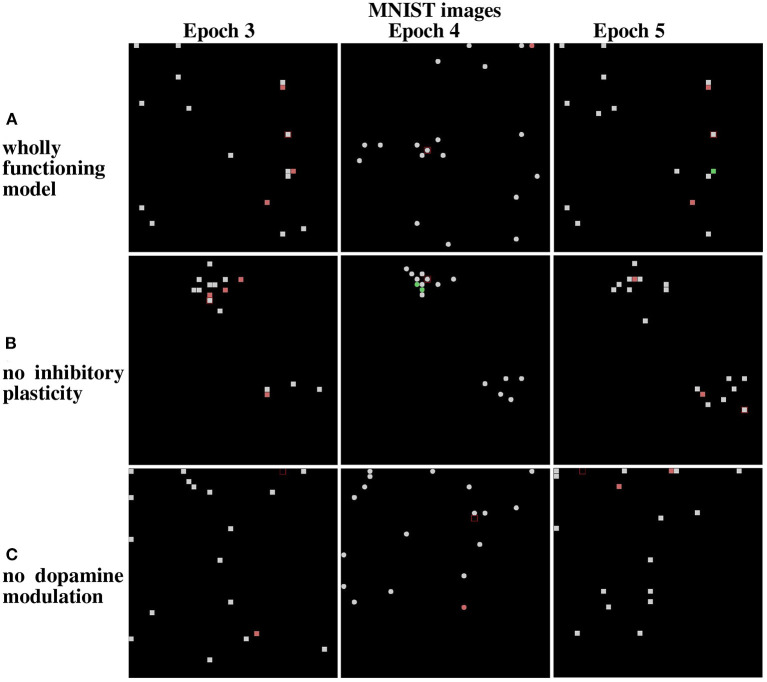
Results from the life-long learning experiment with MNIST images, for the last three epochs that complete the learning. **(A)** Maps refer to model normal operation. **(B)** Maps refer to model operation without modulation of lateral inhibition. **(C)** Maps refer to model operation without dopamine modulation. Epochs 3 and 5 illustrate the results of the first corridor, and Epoch 4 the result of the second corridor. Differently from Figure 12, here the maps corresponding to the epochs 3, 4, and 5 are shown because the model takes more epochs to fully learn the images of the two corridors.

Figure 12, referring to the Lake Como images, and Figure 13, referring to the MNIST images, present the positions of the winning neurons on the neural map, in the last three epochs, in three cases: (a) wholly functioning model; (b) model without inhibitory plasticity; (c) and without novelty-based dopamine modulation. In this experiment, where the model learned the images in the two corridors in sequence, we focused on the stability of the internal representations of the images by looking at the positions of the winner neurons on the neural map. For this purpose, we observed the winning neurons representing images of the first corridor after the agent learned the first corridor (Epoch 1), and then when it revisited the first corridor (Epoch 3) after it learned the second corridor (Epoch 2), to assess the stability of these neurons.

The results show that in the case of Lake Como images in the wholly functioning model, the two mechanisms succeed in protecting the representations after new experiences. In particular, after the second corridor images are learned (epoch 2) there is very little interference in the representations acquired in the first corridor (compare Epoch 1 and Epoch 3, in Figure 12A). As shown in Figure 12B, the removal of the inhibitory plasticity causes a moderate instability effect on the representations of images learned in the first corridor after the second corridor images are learned. Instead, as shown in Figure 12C, the lesion of dopamine modulation has a strong effect, as most of the representations of images learned in the first corridor change after the second corridor images are learned.

We repeated the experiment with the MNIST dataset. In the wholly functioning model, the two mechanisms are quite effective in protecting the representations of the first corridor from the interference of the learning of the second corridor (Figure 13A). The lesion of one of the two mechanisms instead causes a marked instability of the representations of the first corridor (Figures 13B,C).

To check the stability of these results, we repeated all the experiments three times for each operating condition, with three different values of the random generator seed used to generate the initial values of synaptic weights. In Supplementary Table 6 related to the experiment with Lake Como images, and in Supplementary Table 7 related to the experiment with MNIST images, we report the average and standard deviation of the number of distinct neurons having maximum activation with a stable position on the neural map when returning to the first corridor after the second corridor. These data confirm the results that the two mechanisms are very effective in protecting acquired representations from interference.

Overall, the results show that the contextual operation of the two mechanisms succeeds to avoid catastrophic interference. However, the importance of the two mechanisms can vary depending on the features of the observed images. The MNIST images are more similar to each other, due to their simplicity and digit similarity, with respect to the Lake Como naturalistic images, which have richer features and gray tones. The results of the simulations suggest that the more similar the images, the higher the importance of lateral inhibition to avoid the emergence of a dominant population.

## 4. Discussion

### 4.1. Comparison with other models

In this section, we compare our model with other models incorporating bio-inspired mechanisms to face catastrophic forgetting. The first model we consider was proposed by Allred and Roy (2020) which represents a pioneering work in the study of the role of novelty detection as a mechanism protecting networks from catastrophic interference. This model uses spiking neurons, so the resulting network has a higher degree of biological detail than the one presented here. However, the model dynamics contain elements of “global level” control, whereas in our model the control is localized to the neuronal and connectomic level. In particular, in Allred and Roy (2020) the model activation is reset before receiving any input. When a new image is presented, the first neuron to fire receives a temporally enhanced plasticity with the dopaminergic neuron. This mechanism, although biologically implausible, allows rapid learning and protection from forgetting. Instead, our model is more abstract, as it uses firing rate neurons. On the other hand, it is based on continuous functioning and learning (no reset of the network between different images). This led us to develop a model that (a) is able to self-regulate its activation passing from image to image, also based on lateral excitatory connectivity not present in the other model, and (b) uses learning processes that are robust to such dynamics, that is, are not impaired by the passage from image to image. Regarding the solution to the catastrophic interference problem, our model considers not only a dopamine-based mechanism of plasticity of excitatory synapses, as in the model mentioned, but also the plasticity of the lateral inhibitory connections. The latter mechanism is biologically grounded (Udakis et al., 2020) and helps to keep distinct the population codes corresponding to different images.

The work of Udakis et al. (2020) proposes a model, based on data collected with optogenetic procedures, on the role of the plasticity of inhibitory connections in the hippocampus. The model suggests the importance of life-like learning of such lateral inhibitory neuron plasticity, in particular showing that the place fields of neurons tend to change when the model experiences different environments (interference) one after the other when such plasticity is blocked. Our model, which has more detailed connectomics, extends this investigation by considering two different types of images (MNIST and naturalistic images) and by studying how novelty-based and lateral inhibitory plasticity mechanisms affect their learning. This showed that blocking lateral inhibitory plasticity causes interference (non-conservative neural fields) mainly in the case of very similar images (MNIST), while it is of much less importance for dissimilar images (images of Lake Como). Furthermore, we have shown that blocking the novelty-based mechanism facilitates catastrophic interference with both similar and dissimilar images.

We have mentioned in the introduction that the model presented in Kirkpatrick et al. (2017) leads to a segregation of information into different neuron populations. However, this effect was obtained with a machine learning mechanism that freezes the plasticity of the connections that are important for performing previously learned tasks. In contrast, our model uses biologically grounded mechanisms based on dopamine modulation induced by novelty signals, together with the plasticity of the lateral inhibitory connection, leading to the spontaneous emergence of segregation of neural populations encoding different classes. Other hypotheses different from our approach have been proposed on how the brain avoids catastrophic forgetting.

The Complementary Learning Systems theory (McClelland et al., 1995; Kumaran et al., 2016) posits that the hippocampus is capable of fast learning of episodic memories, while the cortex slowly acquires the information stored in the hippocampus through gradual and interleaved learning. This allows the cortex to integrate the new information into the existing cognitive schemes without overwriting them. It has been shown that slow-wave sleep enhances memory consolidation (Born et al., 2006; Marshall et al., 2006; Rasch and Born, 2013) and that during the sleep phase a specifically coordinated replay of recent memory traces occurs in the hippocampus and cortex (Euston et al., 2007; Ji and Wilson, 2007; Maingret et al., 2016). On this basis, slow wave sleep has been proposed as the stage when the hippocampus drives cortical interleaved learning through spontaneous memory reactivation (Rasch and Born, 2013; Kumaran et al., 2016; Maingret et al., 2016). A recent computational model has shown how slow waves can strengthen two competing memory sequences while protecting them from catastrophic forgetting (González et al., 2020). This interesting mechanism can be considered useful for long-term memory consolidation, but it is not sufficient to avoid catastrophic forgetting during memory acquisition in the short term because sleep time cannot follow the learning of every single task, or a few tasks before catastrophic forgetting occurs.

It has also been suggested that adult neurogenesis in the subgranular zone of the dentate gyrus could prevent catastrophic forgetting of episodic memories in the hippocampus (Wiskott et al., 2006; Weisz and Argibay, 2009). New neurons generated in the subgranular zone manifest a lower threshold for LTP, together with the absence of GABAergic inhibition that is instead very strong on the old granule neurons (Wang et al., 2000; Snyder et al., 2001; Schmidt-Hieber et al., 2004). This feature makes the young neurons ideally suited to be recruited in the memory trace encoding new experiences. Some computational models of these phenomena show that, while the network incorporates new information on the young neurons, the previous information is spared because is encoded in the connections of the old neurons (Wiskott et al., 2006; Weisz and Argibay, 2009). However, the role of neurogenesis is still controversial and seems to have different roles in brain functioning depending also on the stages of development and maturation of the adult-newborn neurons. Other studies have shown how neurogenesis can enhance pattern separation during learning (Aimone et al., 2010; Deng et al., 2010; Aimone and Gage, 2011; Sahay et al., 2011; Groves et al., 2013; Agis-Balboa and Fischer, 2014) and how a stimulating environment and training can enhance neurogenesis. However, this type of mechanism, avoidance of catastrophic interference by sparsification and, hence, separation of patterns could also work without adult neurogenesis. Sheer numbers of granule neurons and their strong inhibition may be sufficient (Abrous and Wojtowicz, 2015). Furthermore, although neurogenesis could prevent catastrophic forgetting in murine models there is no evidence that this mechanism is widespread in all mammals. Indeed, the presence of adult neurogenesis in humans is controversial (Kempermann et al., 2018; Duque et al., 2021). While some authors found evidence of newly generated neurons in the adult human hippocampus (Boldrini et al., 2018; Moreno-Jiménez et al., 2019), others came to opposite results (Dennis et al., 2016; Sorrells et al., 2018). A recent *in silico* analysis showed that the neurogenesis-associated markers decline early during development, and suggests that the newly generated cells in the human hippocampus could be part of the glia (Kumar et al., 2020). This prompted us to search for other plausible mechanisms to prevent catastrophic interference beyond neurogenesis.

### 4.2. Limitations and future works

The first limitation of the model concerns the identity of the interneurons simulated. We focused here on lateral inhibition from somatostatin interneurons, leaving out the effect of other interneuronal classes (e.g., feedforward inhibition from parvalbumin interneurons).

A second limitation is in the neuronal connectomics. In the model, we introduced localistic connections between neurons, that decay with distance. In this regard, we were qualitatively inspired by the actual murine connectomic (Bezaire et al., 2016). However, we did not reproduce the connectomics in a quantitative way. Our Mexican hat connectomics resulted in the clustering of neurons coding for similar information, as usually observed in SOMs (Kohonen, 1982). This topological arrangement could change if a different kind of connectomics is used, leading to a sparsification of the neurons encoding similar information (Diehl and Cook, 2015; Hazan et al., 2018). While it is still debated if the hippocampus has a topological organization of the information (França and Monserrat, 2019), computational models employing the actual connectomics of the neuronal populations could shed light on the topic.

Future directions of this work will include the use of different subclasses of interneurons and the study of how their connectomics affect the topology of the SOM.

In the model, we considered binary dopamine (presence/absence) for simplicity (the presence is phasic). If dopamine was continuous rather than binary, we would not expect a substantial qualitative difference, just a slowing down of the learning process. This can be seen from Equation (9), showing that dopamine is used as a multiplicative factor in the learning rule. This issue should be further investigated in future work.

The current model works on the basis of equations that are rather sophisticated as it aims to find sufficient conditions to obtain the results. It would be a valuable effort to check if it is possible to obtain similar results by simplifying the equations. In connection to the previous point, the current model has several parameters: future work might aim to simplify this aspect of the model, so also having more possibility to systematically study the impact of changing them.

## 5. Conclusions

We presented a computational model that can ameliorate catastrophic interference, based on two bio-inspired mechanisms. The first mechanism is a novelty-dependent learning process that mimics dopamine modulation controlled by the ability of the hippocampus to detect new experiences. The model in particular mimics the function of hippocampal units CA1 and Subiculum that together detect novelty/familiarity of images and generate a dopamine-like learning signal dependent on the accumbens/pallidum-ventral tegmental area neural pathway. The core of the novelty detection mechanism is based on the intensity of the activation of the population code inside the pyramidal layer of the hippocampus: with the progress of learning of a certain image, the activation of the population encoding the image grows above a certain threshold, activates the subiculum, and this activates the accumbens/pallidum neuron that in turn inhibits the dopamine outflow of the ventral tegmental area thus stopping learning.

The second mechanism incorporated by the model to address catastrophic interference relies on hippocampal interneurons that ensure the maintenance of homeostasis through inhibitory synaptic plasticity. This in particular increases the inhibition of the winning population code of pyramidal neurons while they are learning. This mechanism has the effect of naturally controlling the level of activation of the winning neurons to prevent them from becoming too reactive to all images, thus prevailing over all other neurons.

The model was tested with two tasks. In a first “catastrophic interference” task the model learned succeeding image sets and the impairment caused by an increasing number of newly learned sets on previous experinece measured. In a second more ecological “life-long learning” task the model fully learned a large set of images and the stability of the learned codes was measured after learning a second large image set. The two tasks used two different types of images, the first one formed by artificial images of numeric digits (MNIST) and a second one from a naturalistic scenario (Lake Como landscape).

The results showed that the two biologically inspired mechanisms are able to substantially ameliorate catastrophic interference. Specifically, in the catastrophic interference test, the two mechanisms prevent the system from incurring strong forgetting with MINST images, whereas with the Lake Como images memory is generally a little lower and a little less impaired by the lack of the two mechanisms. In the lifelong learning test, both mechanisms are necessary to avoid instability of previously acquired experiences with MNIST images, whereas dopamine regulation is sufficient to ensure stability with the Lake Como images.

Overall, the model contributes to showing the important synergistic effect that the two mechanisms, previously studied in isolation, have under different conditions involving different learning regimes and different types of images. Moreover, it contributes to proposing a possible implementation of the two mechanisms having a higher biological detail than in previous models (Allred and Roy, 2020) or a higher specification of mechanisms and ability to scale to realistic images than previously done (Udakis et al., 2020). Finally, the results also show how the two mechanisms, implemented within a neural network having a biologically realistic architecture, can ameliorate catastrophic forgetting by decreasing the overlapping of neuronal populations encoding different images, as was previously done with machine learning methods (Kirkpatrick et al., 2017).

## Data availability statement

The original contributions presented in the study are included in the article/Supplementary material, further inquiries can be directed to the corresponding author.

## Author contributions

PA, FC-M, AM, and GB conceived the model, analyzed the results, and wrote and reviewed the paper. PA conducted the simulations. All authors contributed to the article and approved the submitted version.

## Funding

This work has received funding from the European Union's Horizon 2020 Research and Innovation Program under Grant Agreement No. 713010, project GOAL-Robots – Goal-based Open-ended Autonomous Learning Robots.

## Conflict of interest

The authors declare that the research was conducted in the absence of any commercial or financial relationships that could be construed as a potential conflict of interest.

## Publisher's note

All claims expressed in this article are solely those of the authors and do not necessarily represent those of their affiliated organizations, or those of the publisher, the editors and the reviewers. Any product that may be evaluated in this article, or claim that may be made by its manufacturer, is not guaranteed or endorsed by the publisher.
