# Supplementary material for: A neuro-inspired computational model of life-long learning and catastrophic interference, mimicking hippocampus novelty-based dopamine modulation and lateral inhibitory plasticity
Source: Front Comput Neurosci. 2022 Sep 9;16:954847. doi: 10.3389/fncom.2022.954847 (PMC9500484; doi:10.3389/fncom.2022.954847)
Supplement: Supplementary file 1 [file Data_Sheet_1.pdf]

Supplementary Material

0.1 Supplementary Methods: Input pre-processing

For our purpose all the pre-processesing involving the input image entering into the model are represented by the following operations performed on each observed image. The first operation is the clipping of the input image to avoid noise in the lower values and spike in the higher values, for this purpose we used the numpy $clip()$ and $percentile()$ functions:

$$p = clip(XP, percentile(XP, 20), percentile(XP, imgclip)) \tag{S1}$$

where XP is the input image, p is the clipped image, and the parameter $imgclip$ is equal to 90 for Como images or 95 for MNIST images.

The method for contrast enhancement is a simple linear stretching according to the formula:

$$p' = 255 * (p - img.min())/img.ptp() + min \tag{S2}$$

where p is the clipped image and p' the contrasted image, $img.min()$ and $img.ptp()$ are respectively the minimum value and the range of the values in the input image, and min is minimum desired value in the output image (equal to 0 for Como images or 50 for MNIST images).

Finally, the contrasted image p' is normalized using the L_2 norm to produce for each image pixel the values corresponding to the activation of the input neurons going to the excitatory synaptic connection with the pyramidal neurons.

0.2 Tables

Table S1. Parameters used for the connectivity Gaussian functions (see text).

Layer	s_1	s_2
exc-exc	0.1	
exc-inh	0.1	
inh-exc	0.053	0.002

Table S2. Number of neurons used in simulation for each layer.

Layer	9 : 1 ratio
Input	$40 \times 40 = 1600$
CA1 Pyramidal	$40 \times 40 = 1600$
CA1 Interneuron	$13 \times 13 = 169$
Subiculum	$40 \times 40 = 1600$
Accumbens/Pallidum	1
VTA	1

Table S3. Parameters of neuron equations.

Layer	τ	θ^+ (Activ. threshold)
Input	1	0
CA1 Pyramidal	2	0
CA1 Interneuron	2	0
Subiculum	1	0.92
Accumbens/Pallidum	1	0
VTA	1	0

Table S4. Parameters for CA1 Pyramidal neuron learning rules.

Experiment setup	θ^- (LTP threshold)	h	δ_1	δ_2	L	rep	λ (Noise Lev.)
MNIST, interference	0.37	0.94	1.5	0.0003	0.2	0	0.02
MNIST, corridors	0.445	0.982	3.0	0.01	0.17	1	0.3
Como, interference	0.37	0.94	1.5	0.0003	0.1	0	0.9
Como, corridors	0.43	0.94	3.0	0.001	0.002	1	0.02

Table S5. Results averaged over three repetitions of the lifelong learning experiments with images of Lake Como, using different seeds for the random generator of synaptic weights, in the three operating conditions: wholly functioning model; the lesioned model (no dopamine modulation and no lateral inhibitory plasticity); no dopamine (i.e., no excitatory and inhibitory learning).

Functioning	Number of peaks (average)	STD
Wholly functioning	38.7	1.2
No novelty/inhibitory plasticity	31.0	0.8
No dopamine	15.0	3.3

Table S6. Results averaged over three repetitions of the lifelong learning experiments with images of Lake Como - first corridor -, using different seeds for the random generator of synaptic weights, in the three operating conditions: wholly functioning model; no dopamine modulation; no lateral inhibitory plasticity.

Functioning	Number of peaks (average)	STD
Wholly functioning	15.0	1.0
No novelty	11.0	6.2
No inhibitory plasticity	3.7	0.6

Table S7. Results averaged over three repetitions of the lifelong learning experiments with images of MNIST - first corridor -, using different seeds for the random generator of synaptic weights, in the three operating conditions: wholly functioning model; no dopamine modulation; no lateral inhibitory plasticity.

Functioning	Number of peaks (average)	STD
Wholly functioning	10.7	4.0
No novelty	1.7	1.2
No inhibitory plasticity	2.3	0.6

0.3 Figures

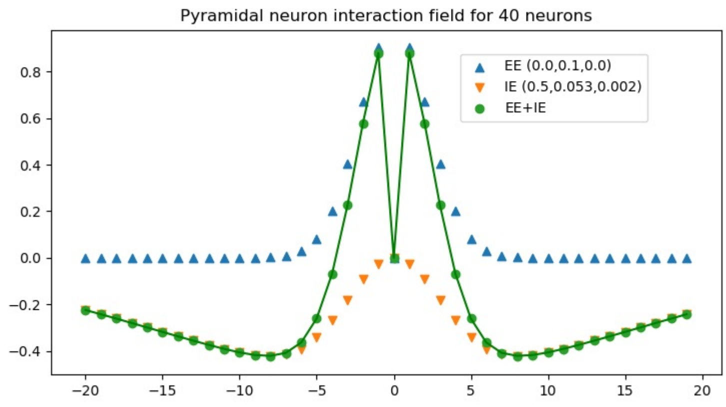

Figure S1. Mexican-hat shaped interaction field of a pyramidal neuron, resulting from the sum of the excitatory (EE) and inhibitory (IE) connection fields.

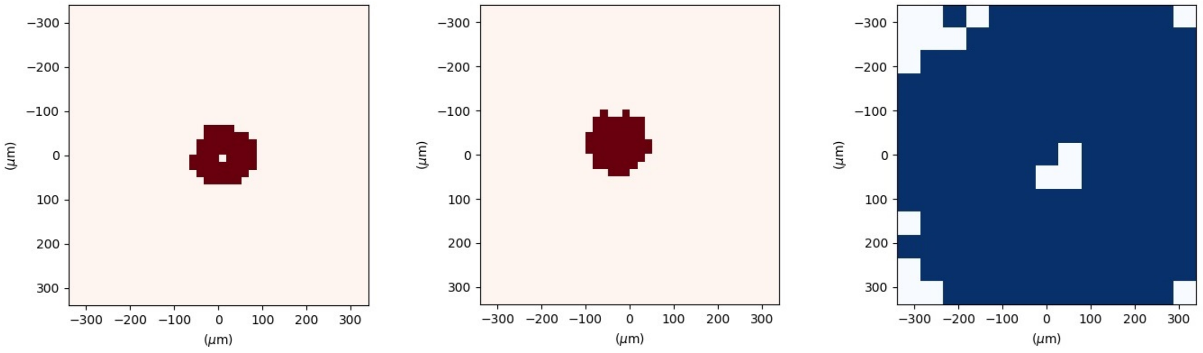

Figure S2. Left: example of the connections of one pyramidal neuron towards the other pyramidal neurons. Centre: example of connections from one pyramidal neuron towards inhibitory neurons. Right: example of connections between one inhibitory neuron towards the pyramidal neurons. The connections plotted are those with weights above the threshold of 0.04.

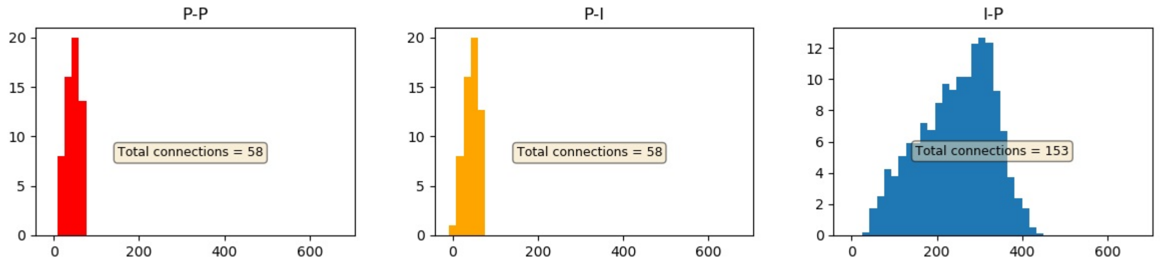

Figure S3. Connection probability by inter soma distance (in μm), measured as connection frequency after the neural network has been generated (data are mediated over the entire network). The left picture reports the pyramidal-to-pyramidal connection frequency (P-P), the middle picture reports the pyramidal-to-interneuron connection frequency (P-I), and the right picture report the the interneuron-to-pyramidal connection frequency (I-P). The ratio between the number of pyramidal neurons to interneurons was set to 9:1 as in CA1. The connections plotted are those with weights above the threshold of 0.04.

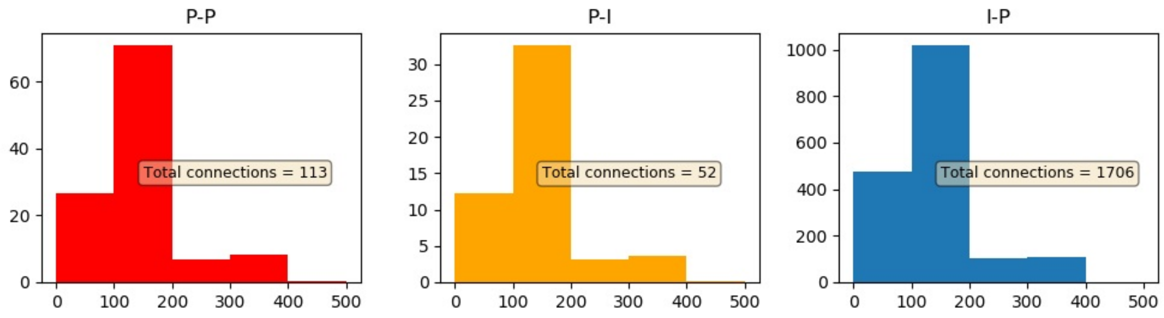

Figure S4. Real CA1: connection probability by inter soma distance (in μm), measured as connection frequency. Data extrapolated from Bezaire et al. (2016). The left picture reports the pyramidal-to-pyramidal connection frequency (P-P), the middle picture reports the pyramidal-to-interneuron connection frequency (P-I), and the right picture reports the interneuron-to-pyramidal connection frequency (I-P).
